# The staphylococcal inhibitory protein SPIN binds to human myeloperoxidase with picomolar affinity but only dampens halide oxidation

**DOI:** 10.1016/j.jbc.2022.102514

**Published:** 2022-09-21

**Authors:** Urban Leitgeb, Paul G. Furtmüller, Stefan Hofbauer, Jose A. Brito, Christian Obinger, Vera Pfanzagl

**Affiliations:** 1 University of Natural Resources and Life Sciences, Vienna, Department of Chemistry, Institute of Biochemistry, Vienna, Austria; 2 Universidade Nova de Lisboa, Instituto de Tecnologia Química e Biológica António Xavier, Oeiras, Portugal

**Keywords:** myeloperoxidase, SPIN, *Staphylococcus aureus*, inhibition mechanism, protein-based inhibitor, protein–protein interaction, halogenation activity, neutrophil, thermodynamics, DSC, differential scanning calorimetry, ITC, isothermal titration calorimetry, MCD, monochlorodimedone, MPO, myeloperoxidase, PDB, Protein Data Bank, SEC, size-exclusion chromatography, SPR, surface plasmon resonance

## Abstract

The heme enzyme myeloperoxidase (MPO) is one of the key players in the neutrophil-mediated killing of invading pathogens as part of the innate immune system. MPO generates antimicrobial oxidants, which indiscriminately and effectively kill phagocytosed pathogens. *Staphylococcus aureus*, however, is able to escape this fate, in part by secreting a small protein called SPIN (Staphylococcal Peroxidase Inhibitor), which specifically targets and inhibits MPO in a structurally complex manner. Here, we present the first crystal structures of the complex of SPIN-*aureus* and a truncated version (SPIN-*truncated*) with mature dimeric leukocyte MPO. We unravel the contributions of the two domains to the kinetics and thermodynamics of SPIN-*aureus* binding to MPO by using a broad array of complementary biochemical and biophysical methods. The C-terminal “recognition” domain is shown to mediate specific binding to MPO, while interaction of the N-terminal “inhibitory” domain is guided mainly by hydrophobic effects and thus is less sequence dependent. We found that inhibition of MPO is achieved by reducing substrate migration, but SPIN-*aureus* cannot completely block MPO activity. Its’ effectiveness is inversely related to substrate size, with no discernible dependence on other factors. Thus, SPIN-*aureus* is an extremely high-affinity inhibitor and highly efficient for substrates larger than halogens. As aberrant MPO activity is implicated in a number of chronic inflammatory diseases, SPIN-*aureus* is the first promising protein inhibitor for specific inhibition of human MPO.

Phagocytosis by neutrophils and subsequent killing of invading pathogens is a highly effective line of defense of the innate immune system (1, 2, 3). Upon activation, neutrophils phagocytose bacteria and release antimicrobial proteins and peptides from subcellular granules into the nascent phagosomal compartment. The heme enzyme myeloperoxidase (MPO) is the most abundant antimicrobial protein and contributes significantly to oxidative killing of pathogens through hydrogen peroxide (H_2_O_2_) mediated formation of highly reactive oxidants. Its most important products are hypochlorous (HOCl) and hypothiocyanous (HOSCN) acid (4, 5, 6) but it can oxidize a range of other physiologically relevant molecules such as bromide, nitrite, phenols, or sulphide (6, 7, 8, 9, 10, 11, 12). However, MPO activity is a two-sided sword as the generated oxidants will indiscriminately attack surrounding biomolecules (13, 14). Thus, MPO is both a key enzyme in host defense and a causative agent in inflammatory diseases, if it is active outside the phagosome. It has therefore attracted considerable attention for the development of therapeutically useful MPO inhibitors (15, 16, 17, 18).

Interestingly, a few pathogens have developed strategies to escape MPO-mediated killing. The most successful human pathogen in combating neutrophil-mediated killing is *Staphylococcus aureus* (*S. aureus*), a bacterium that has evolved strategies against every aspect of host defense (19). In 2017, de Jong *et al*. were able to identify a small 8 kDa protein that has evolved to specifically bind and inhibit human MPO (20). This protein, called SPIN—Staphylococcal Peroxidase Inhibitor—is unique to the *Staphylococcus* genus and is the first known protein-based specific inhibitor of MPO (17, 18, 21).

De Jong *et al*. (2017) first solved the structure of the complex of recombinant monomeric heme-free MPO (rMPO) and SPIN from *S. aureus* (SPIN-*aureus*). The X-ray structure shows that SPIN is composed of two distinct domains. The 6.3 kDa C-terminal alpha-helical domain binds close to the active site. The N-terminal peptide domain is inserted into the substrate channel. Studies using truncations and mutations identified the C-terminal domain as the binding domain responsible for recognition of MPO, while the N-terminal domain and a conserved HDD-motif are required for inhibition. De Jong *et al*. described the mode of inhibition as a “molecular plug” that prevents substrates from entering the active site (22).

Based on the crystal structure, it can be assumed that the two domains bind consecutively. Importantly, the N-terminal domain changes from an unstructured loop in solution to the ß-hairpin domain in the course of complex formation with MPO (22). This resembles the “folding-upon-binding” mechanism proposed for several intrinsically disordered proteins (23, 24, 25). Surprisingly, the contribution of the N-terminal peptide to the overall affinity is reported to be rather small (22) although it amounts to half of the SPIN-MPO surface area and contains 7 out of 17 bond-forming residues (21). This contradicts the general observation that protein–protein interactions are typically governed by favorable enthalpic contributions and protein complexes with higher affinities exhibit large interface areas, a higher number of hydrogen bonds, and high geometric complementarity (26, 27). Clearly, binding and inhibition of MPO by SPIN are closely linked. Therefore, understanding the inhibitory capacity of SPIN—an aspect both vital for *S. aureus* and interesting for MPO inhibitor design—requires understanding not only of the kinetics and thermodynamic driving forces of SPIN binding and complex formation but also of the effect on the activity of MPO including ligand binding and the individual reaction steps of catalyzed redox reactions.

In this article, we present the first crystal structures of the complex of SPIN-*aureus* and a truncated version (SPIN-*truncated*) with mature dimeric leukocyte MPO showing full heme occupancy (Fig. 1). SPIN-*truncated* contains neither the N-terminal domain nor the HDD motif. We could unravel the contribution of the two domains to the kinetics and thermodynamics of SPIN binding to MPO. We demonstrate a strong impact of the N-terminal domain to the overall affinity, which is in contrast to the published data. We can show that although the SPIN N-terminal domain reaches the active site to a certain extent, there is no interaction with the heme cofactor. Moreover, we analyzed the inhibition kinetics and can show that this “molecular plug” (as reported in (22)) is not tight enough for the smaller biological substrates of MPO, such as hydrogen peroxide or halides (6), whose Van der Waals volumes are not significantly larger than that of water http://www.chemaxon.com. Finally, we discuss the structure–function mechanism of MPO inhibition by SPIN with respect to the individual (redox) reaction steps of MPO (28).

**Figure 1:**
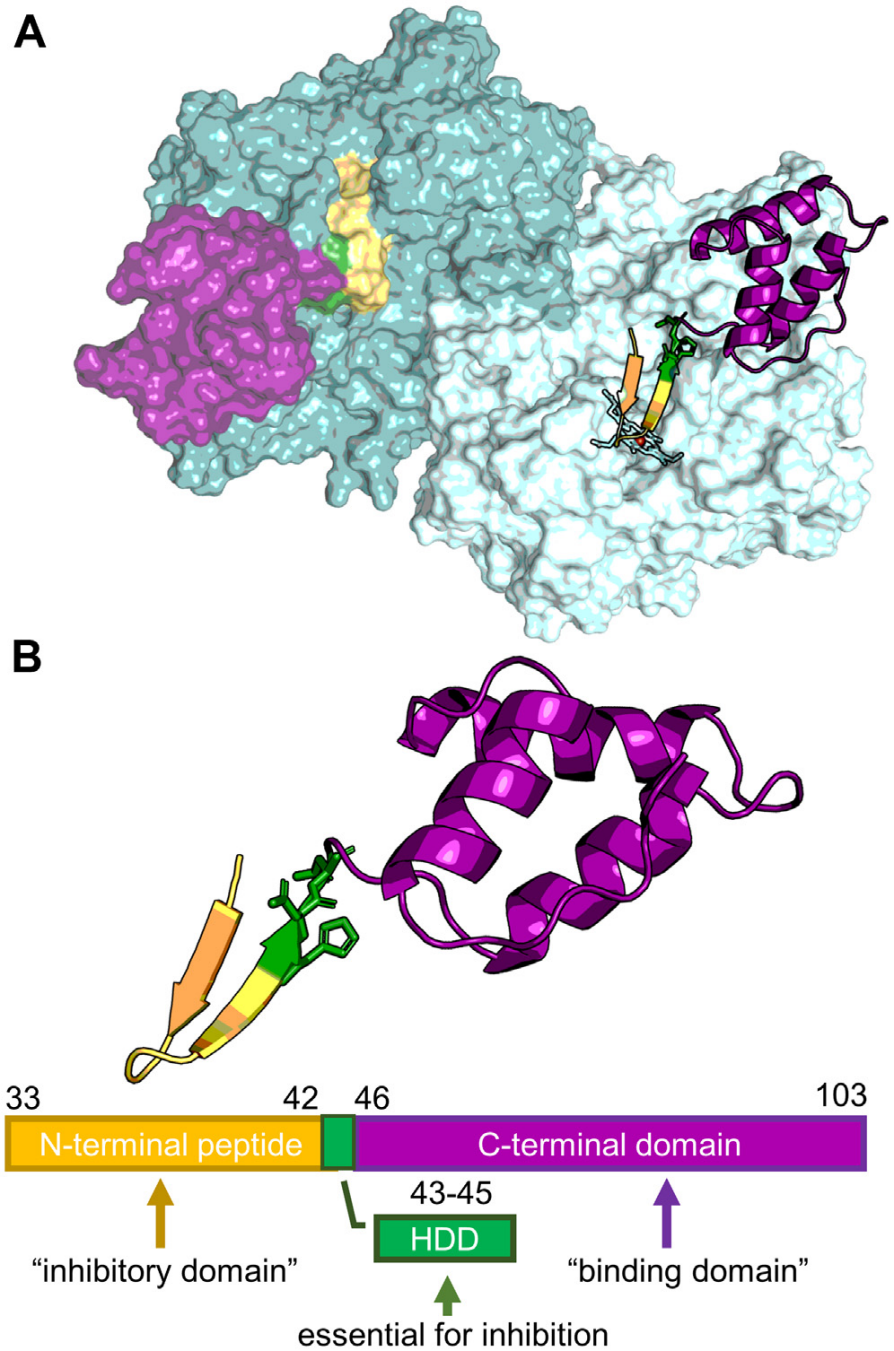
**Architecture of SPIN-*aureus* and crystal structure of the MPO–SPIN-*aureus* complex (PDB code:**7QZR**).***A*, surface representation of mature dimeric MPO (*light* and *dark cyan*) with SPIN-*aureus* shown either as surface or as cartoon representation and colored according to (*B*). The heme cofactor in subunit 2 is shown as a stick representation. *B*, cartoon representation of the SPIN-*aureus* structure in the complex. The N-terminal peptide is colored in *gold*, the HDD-motif is shown as *green sticks*, and the C-terminal domain is shown in *magenta*. MPO, myeloperoxidase.

## Results

### The crystal structure of native human leukocyte MPO in complex with SPIN-*aureus* suggests a split binding interface

As described previously, de Jong *et al*. solved the crystal structure of recombinant monomeric MPO (rMPO) in complex with SPIN-*aureus*. As rMPO differs in several aspects from the native protein, we decided to obtain a cocrystal structure of native human MPO with SPIN-*aureus* (Fig. 1). While rMPO is a single chain monomer with low heme incorporation (29), native human MPO is a ∼150 kDa dimer with each subunit consisting of a heavy and a light chain formed by posttranslational modifications, which are linked by disulfide bridges (30, 31). The heme cofactor is autocatalytically modified and covalently linked to the protein *via* two ester and one sulfonium linkage. Additionally, native MPO is heavily glycosylated at seven N-glycosylation sites (32), with one site in close proximity to the binding site of SPIN-*aureus*.

**Figure 2:**
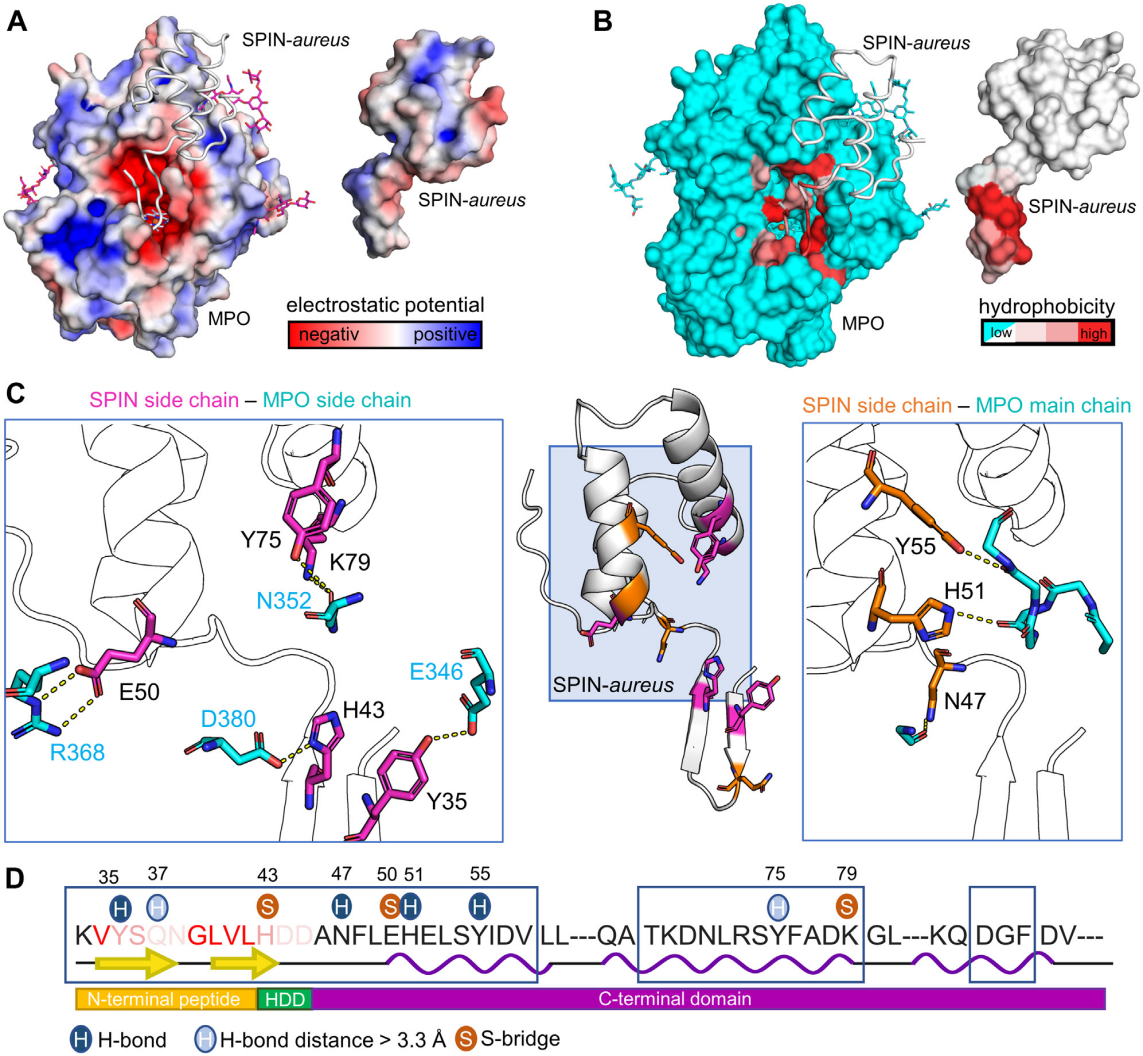
**The interface between mature dimeric human MPO and SPIN-*aureus* (PDB**7QZR**).***A*, electrostatic surface potential (calculated by ABPS) of MPO (left, SPIN-*aureus* depicted as *white loops*) showing the negative potential of the active side (*red*), which is not matched by SPIN-*aureus* (*right*). *B*, hydrophobicity of the active site of MPO (left) and of the N terminus of SPIN-*aureus* (right) colored according to the Eisenberg hydrophobicity scale. The remaining surface is colored *cyan* (MPO) or *white* (SPIN-*aureus*). *C*, hydrogen bonds and salt bridges between side chains of SPIN-*aureus* and side chains (*left*) or the backbone (*right*) of MPO. The central panel depicts interactions between residues of SPIN-*aureus* with side chain residues of MPO in *magenta* and with main chain residues of MPO in *orange*. *D*, interface sequence (*boxed residues*) of SPIN-*aureus* with secondary structure elements and the SPIN domains illustrated below. The color code of the N-terminal residues reflects their hydrophobicity. Interacting residues are highlighted with “*blue circled* H” for hydrogen bonds or “*orange circled* S” for salt bridges. MPO, myeloperoxidase; PDB, Protein Data Bank.

We obtained single crystals of the complex of human native MPO with SPIN-*aureus* that diffracted to a resolution of 2.18 Å and solved the structure to R_work_/R_free_ of 0.20/0.24 (Protein Data Bank [PDB] code: 7QZR). The MPO-SPIN-*aureus* complex crystalized in space group P4_3_2_1_2_1_ with one MPO dimer and two SPIN-*aureus* monomers per asymmetric unit. Overall the crystal structure of the MPO–SPIN-*aureus* complex is similar to the previously solved complex with rMPO (Fig. S1). Superimposition of the structures gave an rmsd of 0.27. The C-terminal globular “recognition” domain binds close to the active site access channel while the N-terminal “inhibitory” domain forms a ß-hairpin motive inserted in the active site (Figs. 2 and S1). The C-terminal domain contributes 680 Å^2^ (45%) and the N-terminal domain contributes 880 Å^2^ (55 %) to the overall interface area (1500 Å^2^). We did not find any interaction with the resolved sugar moieties at Asn355, suggesting that SPIN-*aureus* binding is likely not influenced by the divergent glycosylation patterns found in MPO (33). Gln37, the SPIN-*aureus* residue closest to the heme cofactor, is 3.2 Å away from O_1D_ of the heme propionate, a distance where a weak interaction is possible. Importantly, no direct ligation of the heme iron is observed. This is reflected by the unaltered UV-visible and electronic CD spectra obtained in solution (Fig. S2).

We next analyzed the protein–protein interfaces with regard to (i) electrostatic surface potential (Fig. 2*A*), (ii) hydrophobicity (Fig. 2*B*), and (iii) specific interactions between side chains of SPIN-*aureus* and side chain and main chain atoms of MPO (Fig. 2, *C* and *D*). Analysis of the electrostatic surface potential shows that the MPO substrate access channel is highly negatively charged (Fig. 2*A*). However, no complementarily charged interface can be found in the N-terminal domain of SPIN-*aureus*. In both subunits, only two residues of the N-terminal domain (Tyr35 and Gln37) form hydrogen bonds with residues of MPO, which is in stark contrast to the seven interactions reported by Ploscariu *et al*. in 2018 (21). The reported salt bridge of the nonconserved Lys33 could form. Lys33 is, however, not well resolved, which suggests a high degree of flexibility and contradicts a stable salt bridge. His43 is the only residue of the conserved HDD-motif—reportedly essential for inhibition—that directly interacts with MPO through a salt bridge with Asp380. The interactions of Asp44 and Asp45 reported for rMPO-SPIN-*aureus* are not present in our structure. In total, we found three salt bridges and only four hydrogen bonds with a distance lower than 3.3 Å in both subunits and two with distances up to 3.5 Å. These are mainly in the interface between the C-terminal domain and MPO (Fig. 2, *C* and *D*), suggesting that in this domain, polar interactions are the dominant driving force behind protein binding. A “hot spot” seems to be the stretch of residues 47 to 55 with four out of nine residues forming polar interactions with MPO.

In addition to shape complementarity and polar interactions, interface hydrophobicity is a key driving force for protein binding. The N-terminal domain of SPIN-*aureus* is unstructured in solution but upon binding adopts a distinct β-sheet structure with a hydrophobic patch (residues 39–42) that has a complementary interface with MPO (Fig. 1*B*). Calculation of the solvation free energy gain (Δi*G*) of the overall cocrystal structure and of the two domain interfaces of SPIN-*aureus* individually (Tables 1 and S1) using PISA demonstrates that the N-terminal SPIN domain–MPO interface has a higher Δi*G* (Δi*G* = -8 kcal/mol) than the C-terminal domain (Δi*G* = −4 kcal/mol). This suggests that hydrophobicity plays an important role in folding and binding of the N-terminal domain.

**Table 1:** PISA interface analysis of the crystal structure of native MPO–SPIN-*aureus* complex (PDB: 7QZR), the recombinant MPO-SPIN-*aureus* complex (PBD: 5UZU), and the MPO-SPIN-*truncated* complex (PDB: 7Z53)

Protein/domain/subunit	Interface area [Å^2^]	ΔiG [kcal/mol]	ΔiG *p*-value	H-bonds (>3.3A)	Salt bridges
SPIN-*aureus* (average)	1434 ± 22	12.2 ± 0.3	0.131 ± 0.03		
Subunit 1 (chains AB & E)	1442	−12.5	0.106	6 (4)	3
Subunit 2 (chains CD & F)	1428	−11.9	0.157	6 (5)	3
C-terminal domain E	660	−4.4	0.283	4 (4)	2
C-terminal domain F	670	−4.1	0.349	4 (4)	2
N-terminal domain E	824	−8.5	0.137	2 (1)	1
N-terminal domain F	803	−8.4	0.163	2 (2)	1
Recombinant MPO (5UZU)	1541.9	−10.7	0.280	18	8
SPIN-*truncated* (average)	653 ± 24	−4.1 ± 0.5	0.35 ± 0.04		
Subunit 1 (chains AB & E)	611	−4.1	0.345	4 (3)	2
Subunit 2 (chains CD & F)	661	−4.1	0.356	4 (3)	2
Subunit 3 (chains GH & K)	675	−4.8	0.317	3 (2)	2
Subunit 4 (chains IJ & L)	652	−3.8	0.42	3 (2)	2
Subunit 5 (chains MN & Q)	634	−4.8	0.329	4 (4)	2
Subunit 6 (chains OP & R)	638	−3.5	0.316	4 (3)	2
Subunit 7 (chains ST & W)	676	−4.1	0.343	4 (3)	2
Subunit 8 (chains UV & X)	676	−3.9	0.363	4 (4)	2

Next, we solved the crystal structure of human MPO in complex with a truncated version of SPIN-*aureus*. SPIN-*truncated* contains neither the N-terminal domain nor the HDD motif, which are both essential for inhibition. The cocrystal diffracted to a final resolution of 2.18 Å and R_work_/R_free_ of 0.20 and 0.24. MPO-SPIN-*truncated* crystallized in space group P2_1_22_1_ with four heterotetramers (one dimer MPO bound by two monomers SPIN-*truncated*) per asymmetric unit. PISA analysis of the interface properties suggests that the interface is similar to that of the SPIN-*aureus* C-terminal domain (Tables 1 and S1). Additionally, all hydrogen bonds and salt bridges that were found in the MPO-SPIN-*aureus* cocrystal structure are also present in the MPO-SPIN-*truncated* structure (Table 2). The rmsd of superimpositions of the eight SPIN-*truncated* molecules with the C-terminal domain of SPIN*-aureus* (chain E) were between 0.25 and 0.30. Overall, the crystal structure suggests that SPIN-*truncated* is a good model to understand the initial recognition and binding of the C-terminal domain to MPO (Fig. S1).

**Table 2:** Residues involved in hydrogen bonds and salt bridges between SPIN-*aureus* or SPIN-*truncated* and leukocyte MPO, interacting atoms and interatomic distances in Å between donor and acceptor atoms

Residues SPIN/MPO	Atoms involved	Interaction type	SPIN-*aureus*		SPIN-*truncated*							
			E - AB	F - CD	E - AB	F - CD	K - GH	L - IJ	Q - MN	R - OP	W - ST	X - UV
Hydrogen bonds												
TYR 35/GLU 346	OH…OE2	SC-SC	3.00	2.56								
GLN 37/THR 266	NE2…O	SC-MC	3.44	3.14								
ASN 47/ASN 381	ND2…O	SC-MC	2.82	2.87	3.26	2.88			3.10	3.08	3.16	2.92
HIS 51/ASN 352	NE2…O	SC-MC	3.10	2.93	3.09	2.99	3.11	3.05	3.07	3.12	3.07	3.02
TYR 55/ARG 354	OH…O	SC-MC	2.98	3.12	3.08	2.99	3.14	3.03	3.12	3.12	2.93	3.07
TYR 75/ASN 352^a^	OH…OD1	SC-SC	3.42	3.54	3.49	3.40	3.31	3.63	3.25	3.57	3.38	3.18
Salt bridges												
HIS 43/ASP 380^b^	OE1…NH1	SC-SC	2.62	2.72								
GLU 50/ARG 368^b^	OE2…NE	SC-SC	2.99	3.20	2.77	2.82	2.87	2.67	2.63	3.20	2.87	2.86
	OE1...NH2	SC-SC	3.13	3.05	3.38							
LYS 79/ASN 352	NZ…OD1	SC-SC	2.71	2.78	2.77	3.03	3.11	3.45	3.42	3.38	3.19	3.76
Heme												
GLN 37/HEME	NE2…OD1	SC-Prop	3.17	3.21								

a Interactions found only with PISA.

b Interactions found in both PISA and Chimera.

### Solvation of the interface area of the N-terminal domain of SPIN*-aureus* with MPO and of the substrate-binding sites

**Figure 3:**
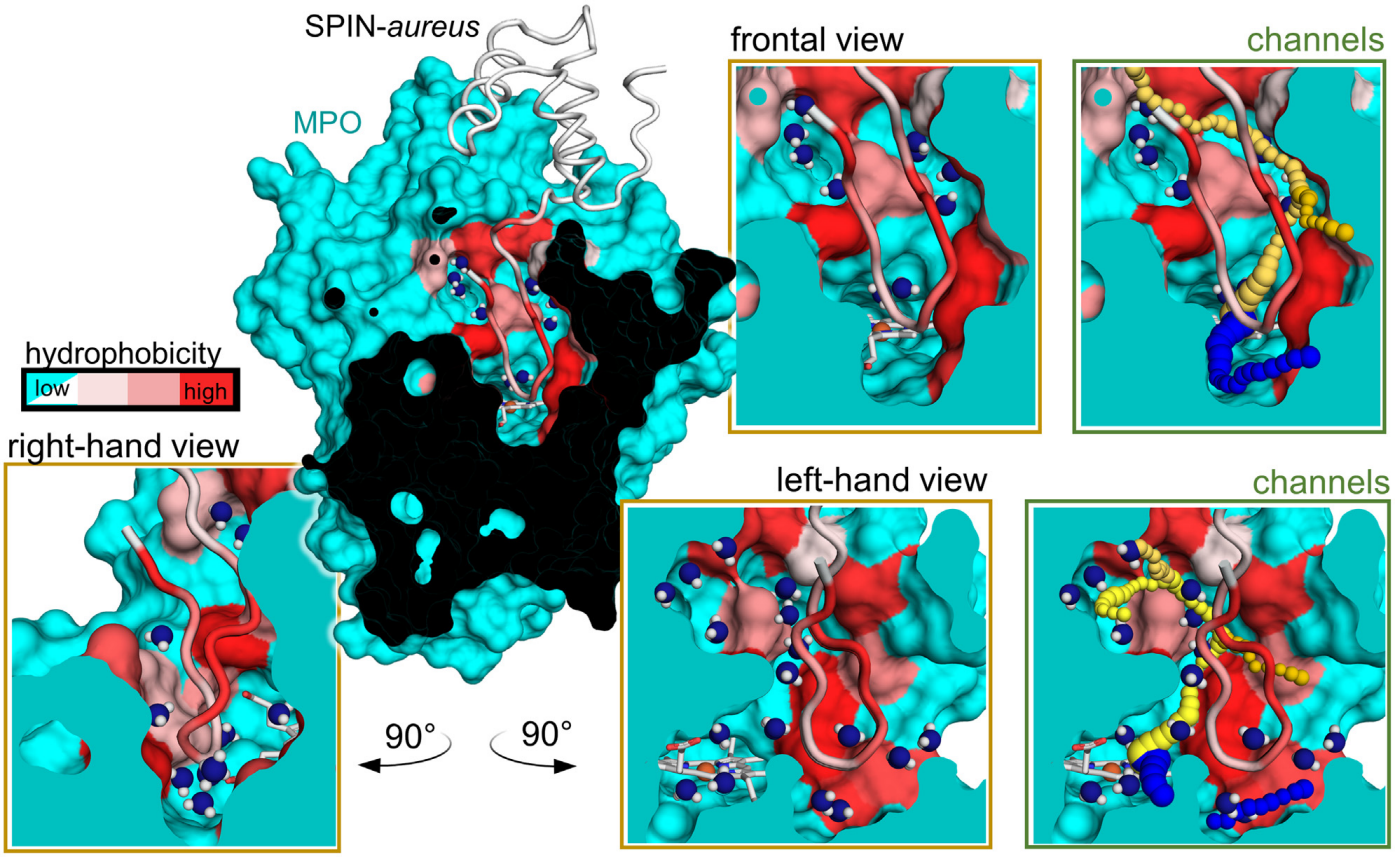
**Water pockets and channels present after SPIN-*aureus* binding in the MPO substrate channel (PDB**7QZR**).** Sliced representation of the MPO–SPIN-*aureus* cocrystal structure in full (center) or of the substrate channel and active site pocket from a frontal, right, and left hand perspective (*yellow boxes*). The water molecules in the active site are shown as *blue spheres*. MPO is shown as surface and SPIN-*aureus* is shown as cartoon putty. The access channel of MPO and the N terminus of SPIN-*aureus* are colored according to hydrophobicity (*cyan* = low hydrophobicity in MPO, *white* = low hydrophobicity in SPIN-*aureus*). Potential access channels (*green boxes*) to the heme cofactor (*white stick* representation) were calculated by Caver3.0 and are shown in the frontal and left hand view as *blue* or *yellow*-*orange spheres*. MPO, myeloperoxidase; PDB, Protein Data Bank.

In addition to formation of electrostatic protein–protein interactions, binding of the N-terminal domain includes displacement of solvent water in the substrate channel. Interestingly, we found that large areas of the MPO access channel, corresponding to the less hydrophobic regions in the interface between MPO and SPIN-*aureus*, contained crystal waters (8 within 5 Å of the N-terminal domain). This includes the positions immediately surrounding the heme cofactor (Fig. 3, *yellow boxes*). To assess accessibility to the heme cavity in the MPO-SPIN-*aureus* complex, we calculated potential access routes using Caver3.0 (https://www.caver.cz/) (34). Two main potential access routes with divergent ends exist (Fig. 3 *green box*, *blue* and *yellow-orange*, Fig. S3). The calculated bottleneck radii were small with 1.1 to 0.9 Å (Table S2), in line with the proposed plug mechanism. SPIN-*aureus* residues framing the bottleneck were Asn38 in the most likely tunnel (*blue tunnel*, Fig. 3) or the hydrophobic patch residues 39 to 42 and His43 (*yellow-orange tunnels*, Fig. 3).

**Figure 4:**
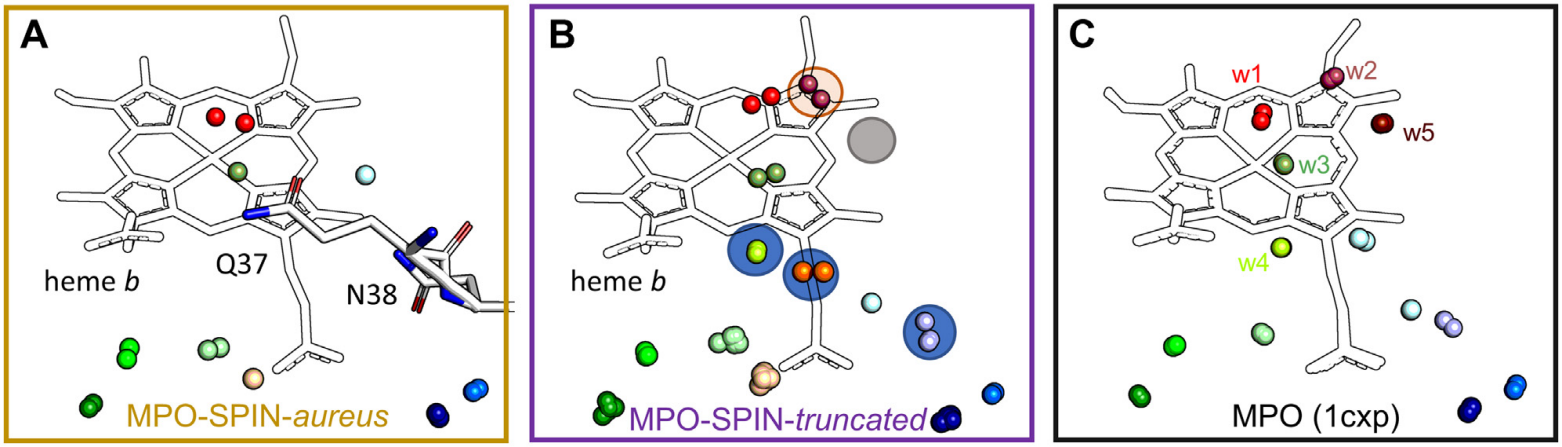
**Threepartially conserved crystal waters are replaced by SPIN*-aureus*.** Crystal waters in the active site of MPO in the presence (*A*) or absence (*B*) of the N-terminal domain of SPIN-*aureus* compared to the structure of MPO alone (PDB code: 1cxp, *C*). Shown are overlays of all subunits (two in the MPO-SPIN-*aureus* cocrystal (*A*) and eight in the MPO-SPIN-*truncated* cocrystal (*B*)). The waters are colored according to position, *red* shows waters in the vicinity of the heme iron, *green* shows waters surrounding the propionate groups, and *blue* corresponds to waters along the blue access channel shown in Figure 2. Conserved water positions replaced by residues of SPIN*-aureus* are highlighted with *blue circles* in the SPIN-*truncated* cocrystal structure (*B*). The label w1 represents the water at the position where H_2_O_2_ and ligands of the heme iron would bind, w2 & w5 represent the waters at the postulated halogen-binding sites, and w3 & w4 are two highly conserved water positions coorindated by the heme proprionate groups. The heme cofactor is shown as *black outline*; the N-terminal residues (Q37 & N38) of SPIN-*aureus* are shown as *white sticks*. MPO, myeloperoxidase; PDB, Protein Data Bank.

We directly compared the crystal waters in the active site and access channel in the MPO–SPIN*-aureus* complex with the MPO-SPIN-*truncated* cocrystal structure and literature data (30). Importantly, one has to bear in mind that the MPO metal center in all crystals likely represents the ferrous form due to the inevitable photoreduction of the heme iron (III) to iron (II) during data collection (35, 36), which influences solvent organization in the first and second coordination sphere of the iron. Five distinct water positions are described (Fig. 4*C*). These are w1, at the position where H_2_O_2_ and iron ligands would bind, w2 & w5, which are at the postulated halogen binding sites, and two conserved waters at w3 & w4. We did not observe the water positions to be as strictly conserved (Fig. 4) as suggested. We observed waters at positions w1 (*red spheres*) in only two subunits of the MPO-SPIN-*truncated* structure. However, this position is the most affected by the iron oxidation state. The general area of the bromide binding site w2 (*raspberry*, highlighted with *orange cycle*) is occupied in two subunits but without a clearly conserved position and the position of w5 (*brown spheres*, circled in *gray*) is not occupied. For these, the effect of photoreduction can only be speculated on. However, the electron density for Glu408 shows that it is only partially linked in all subunits. If Glu408 is not linked, it appears to be very flexible and the carboxyl group would occupy the space of w5 (31). Therefore, we suggest that the previously described heterogeneity observed in the Glu408 ester linkage is the main reason we do not observe waters at this position. More stably coordinated waters (*green*, *wheat*. and *blue spheres*, Fig. 4*B*) were observed around the propionate groups and along the most likely access channel (Fig. 3, *blue channel*). Three of these coordinated waters need to be actively replaced by SPIN*-aureus* residues Gln37 and Asn38 (highlighted with *blue circles* in Fig. 4). Finally, the crystal waters in the access channel are not clearly conserved and we observed between 1 and 11 water molecules that would overlap with the N-terminal residues of SPIN-*aureus*.

### SPIN-*aureus* binds MPO with picomolar affinity, which is predominantly governed by an extremely low dissociation rate mediated by the N-terminal domain

**Figure 5:**
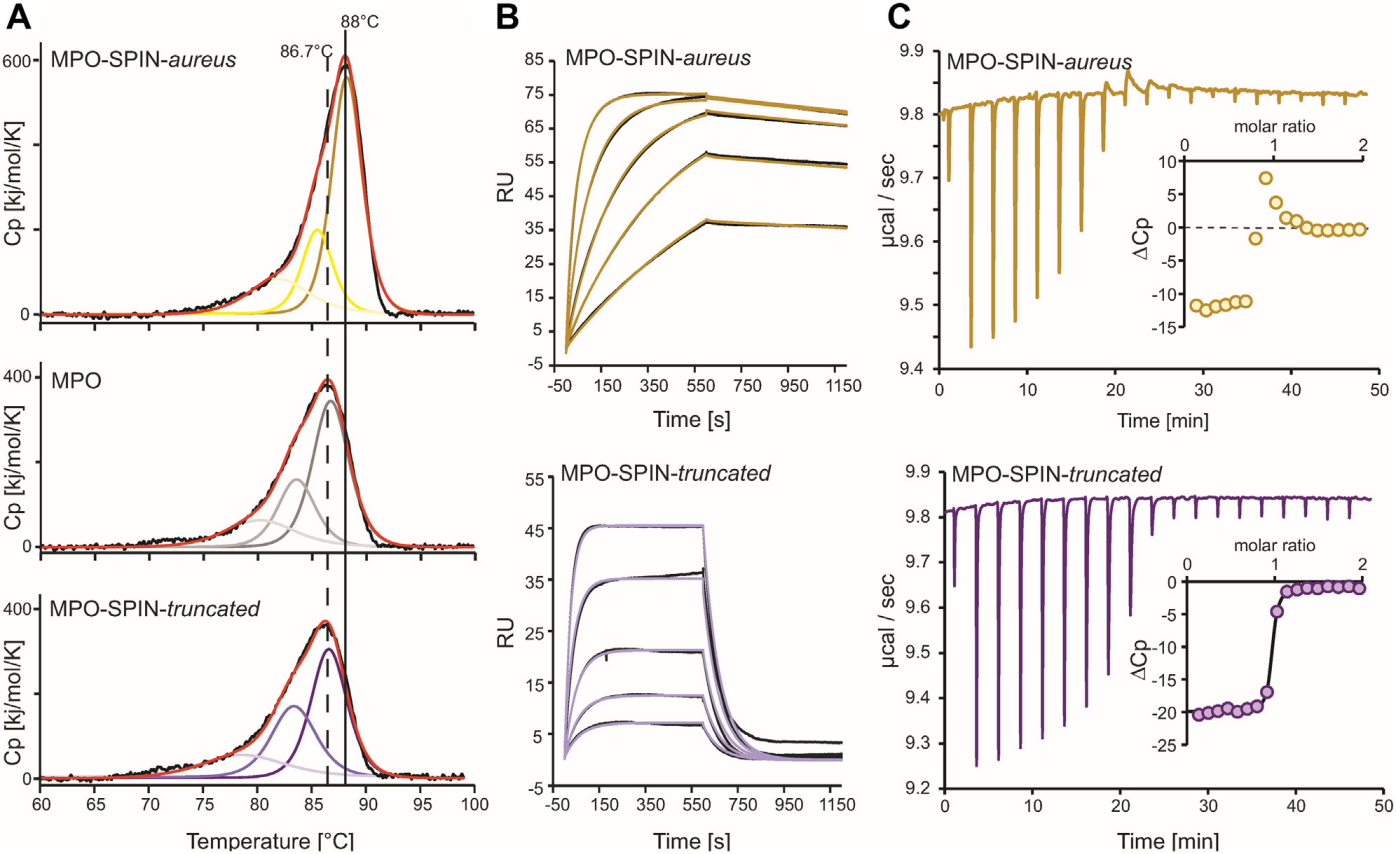
**Kinetics and thermodynamics of binding of SPIN-*aureus* and SPIN-*truncated* to human leukocyte MPO.***A*, DSC thermograms of MPO in complex with SPIN-*aureus* (*top*), MPO alone (*center*), and MPO in complex with SPIN-*truncated* (*bottom*) in 50 mM phosphate buffer at pH 7.4. The corresponding fits using a non–two state transition model are depicted in *red*, the individual transitions in *yellow*, *gray*, or *violet*. The highest *T*_m_ values of each complex are highlighted with *black lines* (full = MPO-SPIN-*aureus* complex, dashed = MPO-SPIN-*truncated* complex). *B*, SPIN-*aureus* (*top*) and SPIN-*truncated* (*bottom*) binding to human MPO monitored by surface plasmon resonance spectroscopy. A 2-fold dilution series (32–2 nM) of recombinant SPIN was injected over biotinylated MPO noncovalently immobilized on a Biotin CAPture chip. The reference-subtracted sensograms (*black traces*) were fitted to a two-state binding model (SPIN-*aureus*, *yellow traces*) or a 1:1 binding model (SPIN-*truncated*, *violet lines*). Representative sensogram series are shown; all experiments were performed in triplicates. *C*, analysis of SPIN-*aureus* (*top*) or SPIN-*truncated* (*bottom*) binding to MPO by isothermal titration calorimetry. Shown are profiles of the heat difference obtained after titration of SPIN-*aureus* or SPIN-*truncated* (150 μM) to MPO (10 μM). Insets show the corresponding integrated injection heats, corrected for the heat of dilution (*circles*). The line shows the best least-squares fit to the one-site binding model. All experiments were performed in triplicates. MPO, myeloperoxidase.

The structural data suggest that the two domains of SPIN-*aureus* interact and bind to MPO through different mechanisms. To better understand and dissect the kinetic and thermodynamic driving forces behind complex formation, we analyzed the kinetic and thermodynamic properties of SPIN-*aureus* with SPIN-*truncated* during binding to MPO. Interestingly, de Jong *et al*. 2018 reported that SPIN-*truncated* binds MPO with the same high affinity (*K*_*D*_ ∼ 10 nM) as SPIN-*aureus* (22). However, the N-terminal domain contributes more than half of the overall interface area, forms a salt-bridge (His43), and is tightly packed within the active site pocket. Both hydrophobic interactions and hydrogen bonds are known to increase protein stability (37), which might be reflected in an increased thermostability. MPO is known to exhibit a high thermostability *per se* with a midterm transition temperature (*T*_m_) of 86.7 °C at pH 7.4 (38). While SPIN-*truncated* did not have a stabilizing effect, we found that the MPO–SPIN-*aureus* complex is significantly stabilized compared to MPO alone in both physiological (pH 7.4: *T*_m_ shift of ∼ 1.5 °C) and acidic pH conditions (pH 5: *T*_m_ shift ∼5 °C) (Table S3 and Fig. 5*A*). The increase in thermostability of the MPO–SPIN-*aureus* complex compared to the truncated version implies a strong interaction, which is in contrast to the minimal effect on binding observed by de Jong *et al* (22).

We therefore tested the binding of SPIN-*aureus* and SPIN-*truncated* by surface plasmon resonance (SPR) using biotinylated native leukocyte MPO immobilized in a two-layer capture strategy *via* hybridization of a DNA-streptavidin conjugate to the sensor surface. Importantly, this setup allows reversible immobilization of the biotinylated protein. We found that SPIN-*truncated* binding to native MPO was comparable to the literature data (*K*_*D*_: 21 ± 8 nM compared to 29 nM, Table 3). Binding of SPIN-*truncated* to MPO was monophasic (Fig. 5*B*) and analysis of the sensograms using a 1:1 binding model allowed calculation of the secondary binding constants (Table 3). Conversely, binding of SPIN-*aureus* was biphasic (Fig. 5*B*) with low to almost no observable dissociation. In accordance with the apparent two domain structure of SPIN*-aureus*, we used a two-state binding model, assuming an initial binding event followed by a conformational change with first order kinetics. For the initial binding event (presumably the C-terminal domain of SPIN-*aureus*), we obtained values similar to SPIN-*truncated* and the reported literature values of SPIN-*aureus* (Table 3), with *k*_on1_ ∼ 6 × 10^5^ M^−1^s^−1^ and *k*_off1_ ∼ 5 × 10^−3^ s^−1^. The association rate constant for the second binding phase (presumably folding and binding of the N-terminal domain) is extremely slow (*k*_on2_ ∼ 0.008 M^−1^s^−1^). Finally, the determined dissociation rate *k*_off2_ of 3.5 × 10^-6^ s^−1^ is at the detection limit of the instrument. Therefore, the *K*_*D*_ value is estimated to be in the lower pM range (*K*_*D*_ ≤ 5 PM), several orders of magnitude lower than reported.

**Table 3:** Binding kinetics of SPIN-*aureus* and SPIN-*truncated* to reversibly immobilized biotinylated native MPO determined by SPR at RT in PBS + 0.1% BSA, 0.05 % Tween 20

Protein	Analyte	*k*_*on*1_ [M^−1^s^−1^]	*k*_*off*1_ [s^−1^]	*k*_*on*2_ [M^−1^s^−1^]	*k*_*off*2_ [s^−1^]	*K*_D_ [nM]
SPIN-*aureus*	native MPO	5.9 ± 2.4 × 10^5^	5.0 ± 1.0 × 10^−3^	0.008 ± 0.001	3.5 ± 2.4 × 10^-6^	0.005 ± 0.006
SPIN-*aureus* (22)	native MPO^a^	5.4 × 10^5^	5.0 × 10^−3^			9.3
SPIN-*aureus* (20)	native MPO^a^	20.1 ± 2.2 × 10^4^	3.2 ± 0.1 × 10^−3^			15.9 ± 2.1
*SPIN-aureus* (22)	rec. MPO^a^	*4.5 × 10*^*5*^	*5.2 × 10*^*−3*^			*11.8*
SPIN-*truncated*	native MPO	1.1 ± 0.6 × 10^5^	1.6 ± 0.2 × 10^−2^			21 ± 8
SPIN-*truncated* (22)	native MPO^a^	6.2 × 10^5^	1.9 × 10^−2^			29.8
SPIN-*truncated* (22)	rec. MPO^a^	4.3 × 10^5^	1.5 × 10^−2^			35.1

Abbreviations: BSA, bovine serum albumin.

The average and SD was calculated from three independent injection series, reported literature values with native or recombinant MPO immobilized by amine coupling.

a immobilization by amine coupling.

To further differentiate the enthalpic and entropic contributions to complex formation, we compared binding of SPIN*-aureus* and SPIN*-truncated* to MPO using isothermal titration calorimetry (ITC) (Fig. 5*C*). Binding of SPIN*-truncated* is enthalpically driven (Δ*H* = −17.4 ± 1.6 kcal/mol) and entropically disfavored (-*T*Δ*S* = 6.0 ± 1.5 kcal/mol). The determined *K*_*D*_ of ∼ 5.9 ± 3.3 nM is within the range of the one obtained from SPR (*K*_*D*_ ∼ 20 nM). Interestingly, we observe a mixed exotherm/endotherm response upon binding of SPIN-*aureus* to MPO (Fig. 5*C*). This is most noticeable in the switch from negative to positive Δ*C*_p_ when a 1:1 ratio is reached in the cell. Likely, the binding of the N-terminal domain causes this endotherm spike, suggesting that it is entropically driven and hydrophobicity is the dominant driving force for binding. Unfortunately, due to the mixed exotherm/endotherm response, we could not determine the thermodynamic properties in more detail.

### Time dependency of MPO inhibition is biphasic

**Figure 6:**
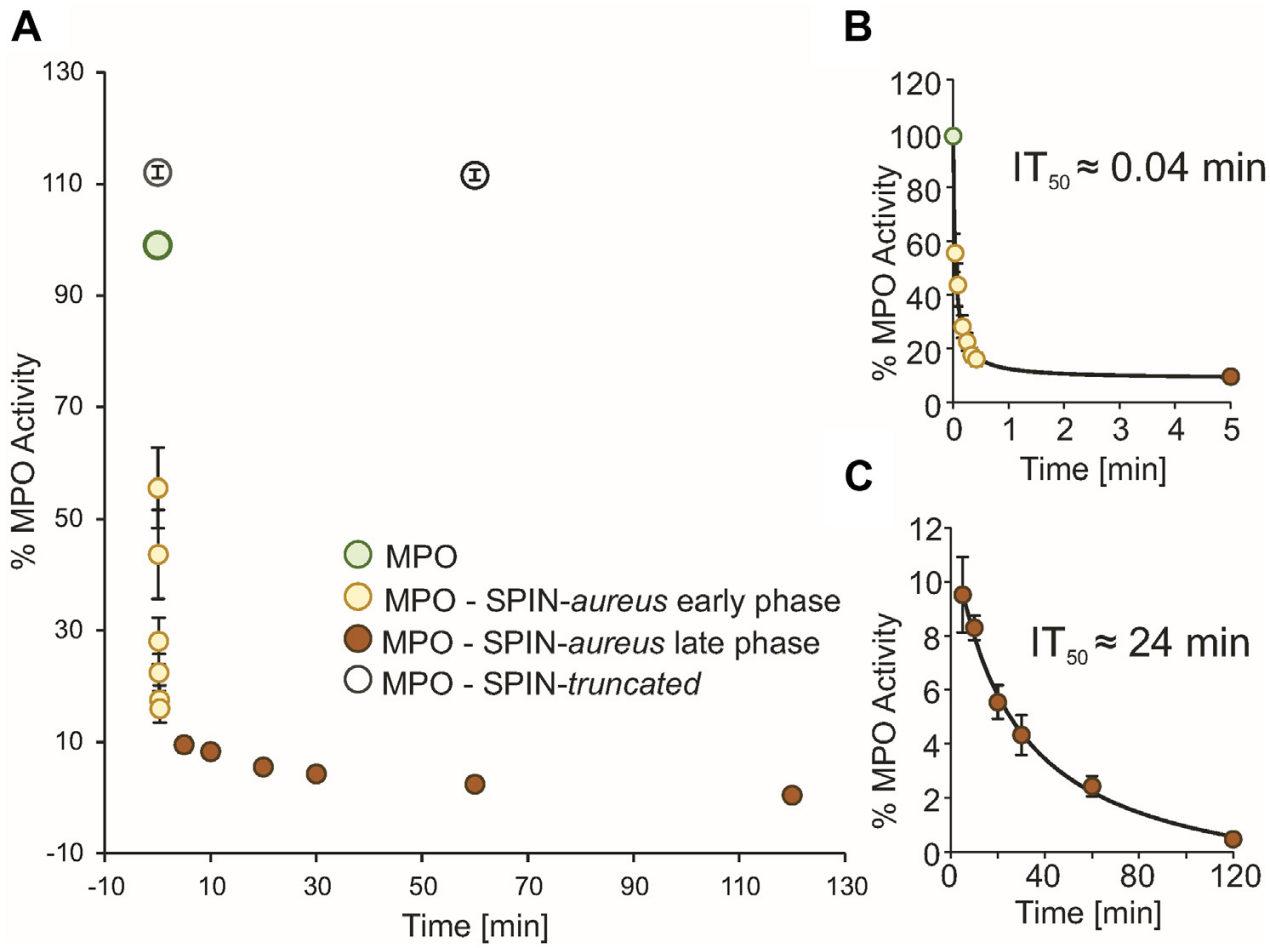
**Inhibition of the bromination activity of human MPO by SPIN-*aureus* is time dependent.***A*, impact of the incubation time of MPO with SPIN-*aureus* on the residual bromination activity. The bromination activity was determined photometrically using the MCD assay, that is, following the decrease in absorbance at 290 nm after addition of 100 μM MPO-SPIN-*aureus* complex to 5 mM bromide and 150 μM H_2_O_2_ in 50 mM phosphate-citrate buffer pH 5. As a control bromination of MPO without SPIN and after incubation with SPIN-*truncated* were measured (*i.e*., 100% activity). *B*, determination of the half maximum inhibition time (IT_50_) of the first phase (*light brown* cycles, incubation time up to 5 min). *Black line*: hyperbolic fit. *C*, determination of the half maximum inhibition time (IT_50_) of the second phase (*light brown* cycles, prolonged incubation times from 5 min to 120 min). *Black line*: hyperbolic fit. All experiments were performed in triplicates and represented as mean (SD). MCD, monochlorodimedone; MPO, myeloperoxidase.

SPR analysis suggests a large time difference between initial binding of MPO by the C-terminal domain of SPIN-*aureus* (*k*_on_ ∼ 10^6^ M^−1^s^−1^) and inhibition upon binding of the N-terminal domain (*k*_on_ = 0.008 M^−1^s^−1^). To test whether and how this two-phase binding affects the inhibition of the enzyme, we measured the inhibition of the bromination activity of MPO preincubated with SPIN*-aureus* for defined time intervals. Figure 6 clearly shows that MPO inhibition is biphasic with an initially fast loss in activity followed by a slower second inhibition phase. Two half maximal inhibition times IT_50_ from separate hyperbolic fits of the two phases (Fig. 6, *B* and *C*) were determined. MPO inhibition in the early phase (incubation times 0–5 min) leads to ∼80% total inhibition with an IT_50_ of 2.4 s, while inhibition up to 99.5% is only achieved after 2 h of incubation (IT_50_ = 24 min).

### Inhibition efficiency of SPIN*-aureus* depends on the molecule size of substrate or ligand

The crystal structure of the complex, as well as in solution data from UV-visible and electronic CD spectroscopies (Fig. S2), clearly show that there are no significant direct interactions between the heme iron and residues of the N-terminal domain of SPIN-*aureus*. As outlined previously, we have found a large number of water molecules still present in the MPO substrate channel and heme pocket even when SPIN-*aureus* is bound. We also observed residual activity even after 2 h of incubation. In principle, the main biological substrates of MPO, that is, H_2_O_2_, chloride, bromide, and thiocyanate (SCN^-^), as well as its reaction products, that is, (pseudo)hypohalous acids, are all relatively small, with Van der Waals radii close to that of water. This also applies to typical heme ligands, which might allow access of these molecules to the heme cavity even in the presence of SPIN-*aureus*. We therefore decided to test the impact of the molecular properties (*i.e*., charge, size, and geometry) of substrates and ligands on the accessibility to the heme iron. We analyzed the reaction of native MPO and MPO–SPIN-*aureus* (equimolar concentration, one-hour pre-incubation) with hydrogen peroxide and the heme ligands cyanide (HCN), nitrite (NO_2_^-^), and sulfide (HS^-^/H_2_S) by using conventional stopped-flow spectroscopy.

**Figure 7:**
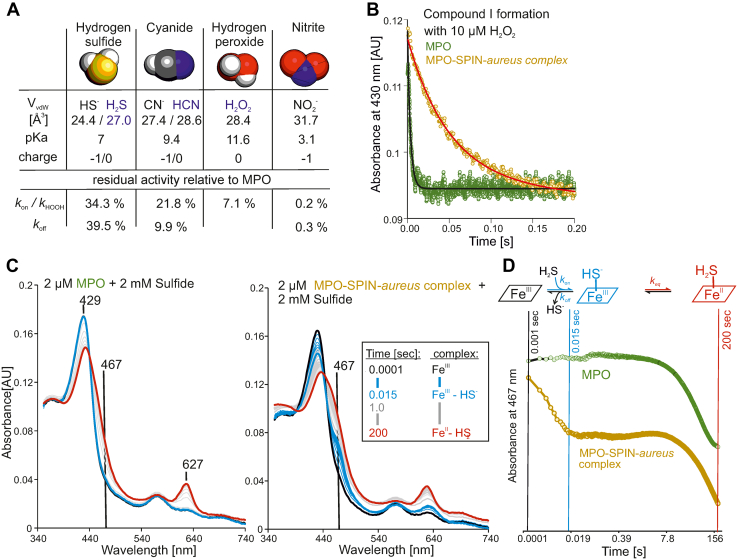
**Compound I formation and ligand binding of MPO and MPO–SPIN-*aureus*.***A*, molecular parameters of the tested ligands and substrates (hydrogen sulfide, cyanide, hydrogen peroxide, and nitrite) and the change in rate constants *k*_on_, *k*_off_, and *k*_HOOH_ relative to free MPO. *B*, compound I formation of MPO (*green*, single exponential fit in *black*) or MPO-SPIN-*aureus* (*yellow*, single exponential fit in *red*) upon addition of 10 μM followed at 430 nm and pH 7. *C*, spectral transitions of MPO and MPO–SPIN-*aureus* complex upon reaction with 2 mM hydrogen sulfide. Spectra are colored according to time after mixing (initial: *black*, up to 0.015 s: *cyan*, up to 200 s: *gray*, last spectrum: *red*). *D*, schematic representation of the reaction of the heme cofactor with hydrogen sulfide and time traces of the reaction of MPO and MPO–SPIN-*aureus* complex at 467 nm. MPO, myeloperoxidase.

Hydrogen peroxide mediates the two-electron oxidation of ferric MPO to the redox intermediate Compound I. This rapid reaction represents the initial step of both the peroxidase and halogenation cycle of MPO and can easily be followed by the decrease in Soret absorbance at 430 nm (Fig. 7*B*) (28, 32). At pH 7.0, the apparent bimolecular rate constant of Compound I formation was determined to be 1.0 × 10^7^ M^−1^ s^−1^ and 7.1 × 10^5^ M^−1^ s^−1^ for native MPO and MPO-SPIN-*aureus*, respectively (Fig. 7*B*). The presence of SPIN-*aureus* had no impact on the spectral transitions accompanying the oxidation of ferric MPO to Compound I (Fig. S4).

Binding of the low-spin ligands cyanide, nitrite, and sulfide can be followed by a red shift of the Soret maximum of high-spin MPO or MPO–SPIN-*aureus* to the Soret maximum of the respective low-spin complexes (28, 32). The obtained kinetic data clearly demonstrate that the limited accessibility due to the presence of SPIN-*aureus* is more pronounced with bigger ligands following the hierarchy sulfide < cyanide (<H_2_O_2_) < nitrite (Fig. 7*A*). The calculated association (*k*_on_) and dissociation rates (*k*_off_) for MPO and MPO–SPIN-*aureus* are summarized in Table 4. In all ligands, both *k*_on_ and the *k*_off_ are reduced to the same extent in the presence of SPIN-*aureus*. The degree of reduction is strictly dependent on the ligand size (native MPO: 100%): ∼30% (sulfide) > 20% (cyanide) > 7% (H_2_O_2_) > 0.2% (nitrite). As a consequence, binding of SPIN-*aureus* had only a small impact on the respective *K*_*D*_ values (Table 4).

**Table 4:** Association and dissociation rate constants and *K*_*D*_ values determined from pre–steady-state kinetics of ligand binding to MPO alone or in complex with SPIN-*aureus*

	MPO			MPO-SPIN-*aureus* complex		
Substrate/Ligand	*k*_on_ [M^−1^s^−1^]	*k*_off_ [s^−1^]	*K*_*D*_ [μM]	*k*_on_ [M^−1^s^−1^]	*k*_off_ [s^−1^]	*K*_*D*_ [μM]
Cyanide	2.4 × 10^6^	2.26	0.95	5.2 × 10^5^	0.22	0.43
Hydrogen sulfide	n.d.	n.d.	n.d.	8.5	2.88	33.9
	4.71	0.011	2.3 × 10^3^	1.62	4.3 × 10^−3^	2.6 × 10^3^
Nitrite	3.0 × 10^6^	54.57	18.33	6.0 × 10^3^	0.15	24.16
	*k*_HOOH_[M^−1^s^−1^]			*k*_HOOH_ [M^−1^s^−1^]		
Hydrogen peroxide	1 × 10^7^			7.1 × 10^5^		

It has to be mentioned that, compared to cyanide or nitrite, sulfide binding to MPO is more complex because it acts as both ligand and reductant. It is a two-step mechanism that includes binding to native MPO followed by the formation of the ferrous protein (39) The overall spectral transitions accompanying this two-step reaction are similar with MPO and MPO–SPIN-*aureus* (Fig. 7*C*). The kinetics, however, are different (Fig. 7*D*). With native MPO, sulfide binding to the ferric protein cannot be detected and only the slower transition to the ferrous MPO is visible (Fig. 7*D*). In the presence of SPIN-*aureus*, the MPO(III)–sulfide complex can be trapped since both *k*_on_ and *k*_off_ are slowed down (blue spectra Fig. 7*C*). An intermediate species with a maximum at 467 nm can be observed during the first 15 ms of the reaction. The second reaction phase, that is, formation of the ferrous form, must be independent of SPIN-*aureus* as it is an equilibrium rate between two forms where the substrate is already in place. Therefore, the determined rate in reduction (∼30% decrease in velocity) can be attributed to sulfide binding. This suggests that sulfide binding to MPO is faster than previously described (*k*_on_ MPO ≥ *k*_on_ MPO-SPIN-*aureus*: 10^4^ M^−1^s^−1^), with a *K*_*D*_ of ∼ 30 μM (39).

Finally, we determined the steady-state kinetic parameters for H_2_O_2_-dependent bromination and chlorination of monochlorodimedone (MCD) to test if the halogen binding site is perturbed by SPIN-*aureus* (Table 5). We found that while *k*_cat_/*K*_M_ for H_2_O_2_ and bromide were both reduced by a factor of ∼100, this was due to a reduction in the turnover number (MPO: *k*_cat_ ∼ 27 s^−1^, MPO-SPIN-*aureus*: *k*_cat_ ∼ 0.7 s^−1^). The same was observed for chloride (MPO: *k*_cat_ ∼ 26 s^−1^, MPO-SPIN-*aureus*: *k*_cat_ ∼ 0.5 s^−1^). As chloride has a smaller ionic radius than bromide, the inhibition by SPIN-*aureus* should be less efficient. Unfortunately, due to the low residual activity of the complex and the corresponding high uncertainty of the kinetic measurements, we can only speculate on this. Importantly, the presence of SPIN-*aureus* had no significant impact on the *K*_*M*_ values. This supports the hypothesis that the binding sites of both hydrogen peroxide and bromide or chloride are not perturbed (Table 5).

**Table 5:** Kinetic parameter (mean ± SD) of MPO bromination and chlorination of MCD without or in complex with SPIN-*aureus*

Kinetic constant	Bromide		Chloride	
	MPO	MPO–SPIN-*aureus* complex	MPO	MPO–SPIN-*aureus* complex
*V*_max_ [μMs^−1^]	2.76 ± 0.85	0.07 ± 0.01	2.64 ± 0.43	0.05 ± 0.01
*K*_M_B (H_2_O_2_) [μM]	26.67 ± 12.31	24.74 ± 6.28	64.04 ± 13.62	133.70 ± 15.52
*K*_M_A (Br/Cl) [μM]	1.32 ± 0.78 × 10^3^	2.44 ± 0.13 × 10^3^	6.31 ± 1.32 × 10^4^	12.87 ± 10.7 × 10^4^
*k*_cat_ [s^−1^]	27.63 ± 8.60	0.69 ± 0.05	26.39 ± 4.31	0.50 ± 0.10
*k*_cat_/K_M_B [M^−1^s^−1^]	1.04 ± 0.30 × 10^6^	2.79 ± 0.96 × 10^4^	4.14 ± 2.09 × 10^5^	3.70 ± 3.34 × 10^3^
*k*_cat_/*K*_M_A [M^−1^s^−1^]	2.10 ± 1.20 × 10^4^	2.83 ± 0.59 × 10^2^	3.90 ± 0.1 × 10^2^	6.4 ± 6.1

## Discussion

*S. aureus* is uniquely inventive in its fight to evade the human innate immune system. Among others, it has evolved the fascinating small protein SPIN-*aureus* that specifically binds and inhibits human MPO. This work aimed at understanding the MPO–SPIN-*aureus* complex in detail in order to elucidate the molecular mechanisms of how SPIN-*aureus* inhibits the generation of antimicrobial oxidants by MPO.

Despite its small size, SPIN-*aureus* has two distinct domains, with the N-terminal domain changing from an unstructured state to a ß-sheet structure during complex formation with MPO. Conformational changes due to protein–protein interactions as well as “folding-upon-binding” mechanisms known for disordered protein regions or intrinsically disordered proteins are often seen in biology but mostly found at surface areas. The N-terminal peptide of SPIN-*aureus*, however, adopts a ß-sheet structure that reaches ∼18 Å into the access channel of MPO, a fact not to be neglected in terms of folding. Folding of the N-terminal domain of SPIN-*aureus* is only induced after molecular recognition of MPO by the globular C-terminal domain that binds close to the substrate access channel. Thus, SPIN-*aureus* binding to MPO follows a distinct sequence of events, that is, (i) binding of the C-terminal recognition domain, followed by (ii) folding, and (iii) insertion of the N-terminal inhibitory domain (or *vice versa*). By comparing SPIN-*aureus* with SPIN-*truncated* as a model for the C-terminal domain, we were able to dissect the thermodynamics and kinetics of these reaction steps and demonstrate that the published *K*_*D*_ for both native and recombinant MPO of 10 nM to 20 nM (which already placed the MPO–SPIN-*aureus* complex among high affinity protein complexes) is too high. This is because it only reflected the binding of the C-terminal globular domain and fully neglected the contribution of the N-terminal domain (Table 3). In this work, we estimate the *K*_*D*_ value of the MPO–SPIN-*aureus* complex to be ∼10^−12^ M or likely is even lower. This places the MPO–SPIN-*aureus* complex close to the highest affinity protein–protein heterocomplexes. For comparison, the streptavidin–biotin complex has a *K*_*D*_ of 10^−15^ M (40). In this case, it is due to a large conformational change and structural adaptation of the N-terminal domain of streptavidin and the concomital low dissociation rate. Unfortunately, it still remains technically challenging to determine such high protein—protein affinities accurately as it is at or beyond the detection limit of most methods.

Binding of the small globular C-terminal domain to MPO is predominantly enthalpically driven (Δ*H* ∼ −17 kcal/mol) and results in a nanomolar affinity. The overall picomolar affinity is only achieved upon the comparably slow binding of the N-terminal domain (*k*_on_ ∼ 0.008 M^−1^s^−1^) and governed by the practically unmeasurable dissociation rate (*k*_off_ = 10^−6^ s^−1^ or even lower). Contrary to the previous published structures with monomeric recombinant MPO, we did not find significant electrostatic interactions between the N-terminal peptide and the heme enzyme. Instead, we identified the hydrophobic GLVL patch preceding the HDD motif that aligns along an equally hydrophobic area in the MPO access channel (Fig. 2). Desolvation of the access channel, which imposes an enthalpic penalty on binding, appears to be limited, as several water-containing cavities remain (Fig. 3). Of all well-coordinated crystal waters present in the X-ray structure of MPO-SPIN-*truncated*, only three are replaced by residues Gln37 and Asn38 of the N-terminal domain (Fig. 4). Interestingly, we observe an endotherm overlaying the binding exotherm in ITC titration experiments only with SPIN-*aureus* (Fig. 5). Therefore, desolvation of the active site, which imposes an enthalpic penalty (Δ*H* > 0) is not compensated by the newly formed electrostatic interactions of the N terminus and HDD motif, as is the case during binding of the C-terminal domain. As binding of the N terminus is enthalpically disfavored, it must be entropically driven. The overall entropy change of this binding step includes both the entropy contributions of the proteins, that is, the cost of forming the MPO–SPIN complex and the conformational entropy cost upon folding of the N-terminal domain, together with solvent entropy changes due to desolvation of the interfaces. As the contribution of the proteins will be unfavorable, the increase in solvent entropy due to release of waters from the active site and from the N-terminal domain into the bulk solvent must be the driving force. An example for entropically driven binding is the class I MHC, where this effect was attributed to the hydrophobic nature of the peptide binding groove (41). Interestingly, a recent study on MHC suggested that interface waters in nonpolar cavities may have a higher entropy than bulk solvent (42). We also observe a number of water molecules in three cavities in the interface between the MPO active site and the N-terminal hydrophobic GLVL patch (Fig. 4), which may contribute in a similar manner.

The crystal structure, as well as in-solution spectroscopic data and the unaltered affinity for all tested ligands, show that SPIN-*aureus* does not directly interact with the heme iron. Additionally, the relatively small change in *K*_*M*_ compared to *k*_cat_ determined for bromide oxidation suggest that the substrate-binding sites for H_2_O_2_ and halogens are not perturbed by SPIN-*aureus*. Thus, it is reasonable to assume that the peculiar redox properties of human MPO (28) and the reduction potentials of its catalytically relevant couples Fe(III)/Fe(II), Compound I/Fe(III), Compound I/Compound II, as well as Compound II/Fe(III) (43, 44, 45), remain unaltered in the MPO–SPIN-*aureus* complex. MPO can therefore still follow the halogenation cycle and catalyze H_2_O_2_-mediated chloride oxidation. However, we found a strong correlation between substrate size and reduction in the apparent second-order rate constants. This will apply to all reactions and is shown here through ligand binding, formation of Compound I, and through steady-state analysis of bromination and chlorination activity. In all cases, SPIN-*aureus* does not completely block the substrate channel, still allowing small biological substrates to pass through, albeit in a slowed-down mode. Thus, inhibition of MPO is solely based on sterically limiting substrate/product shuttling through the access channel. Due to the slow binding rate of the N-terminal domain, MPO inhibition is strongly dependent on time but ultimately SPIN-*aureus* is able to inhibit MPO activity by 99.8% after 2 h of incubation. Nevertheless, inhibition of MPO will always be incomplete and strongly dependent on the size of the electron donor. This means that SPIN-*aureus* is likely more effective against large substrates (SCN^−^) than small ones (Cl^−^) and most probably very effective against typical aromatic one electron donors that are directly oxidized at the heme edge (32).

Taken together, we have provided comprehensive structural, thermodynamic, and kinetic analyses of SPIN-*aureus* binding to and inhibition of mature dimeric human MPO. Specific recognition of MPO is predominantly mediated by the C-terminal domain, while interaction of the N-terminal domain is guided by hydrophobic effects and is thus less sequence dependent. Binding of the N-terminal domain is very slow, provides picomolar total affinity, and does not block access of hydrogen peroxide or halides nor does it disrupt the active site architecture. Several questions still remain to be answered, especially regarding the sequence of events in binding and folding of the N-terminal domain and how this refolding is induced. How *S. aureus* has evolved this unique protein is not known yet. Investigations on the flexibility and malleability of the N terminus are under way. With its picomolar affinity, which is several orders of magnitude lower than the best tested small molecule inhibitors, SPIN-*aureus* is the first truly specific and high affinity inhibitor of MPO (46).

## Experimental procedures

Highly purified dimeric leukocyte MPO of a purity index (*A*_428_/*A*_280_) of at least 0.85 was purchased as lyophilized powder from Planta Natural Products (http://www.planta.at/) and the concentration was determined spectrophotometrically using *ε*_428_ = 91,000 M^−1^ cm^−1^ per heme (47).

### Cloning, expression, and purification of SPIN-*aureus* and SPIN-*truncated*

The codon optimized sequences for SPIN-*aureus* and SPIN-*truncated* were cloned in frame with an His-SUMO expression tag (backbone pSUMO) using the NEBuilder HiFi DNA Assembly Master Mix (NEB # E2621S); PCR amplification of all fragments was done using Q5 High-Fidelity 2× Master Mix (NEB #M0492L) and primers 3′- TGAGGCTCACCGCGAACAGATTGGAGGTAAAGTTTATTCCCAGAAC-5′ and 3′- GTTCTGGGAATAAACTTTACCTCCAATCTGTTCGCGGTGAGCCTCA-5’ (SPIN-aureus), 3′- ACCGCGAACAGATTGGAGGTGCCAATTTCCTGGAGCACG-5′ and 3′- GTTCTGGGAATAAACTTTACCTCCAATCTGTTCGCGGTGAGCCTCA-5’ (SPIN-truncated). and 3′-TGATGACTCGAGCACCACCACCACC-5′ and 3′- GTTCTGGGAATAAACTTTACCTCCAATCTGTTCGCGGTGAGCCTCA-5’ (pSUMO backbone). For protein expression, all plasmids were transformed in *Escherichia coli* BL21 C41 and cultivated in LB medium with kanamycin (final concentration 100 μg mL^−1^) in shaker flasks (180 rpm, 37 °C) until OD_600_ of ∼0.6 to 0.8, followed by reduction of the temperature to 16 °C and induction using IPTG (final concentration 0.5 mM) and growth was continued at 16 °C and 180 rpm overnight.

Protein purification was performed by His-affinity purification, SUMO protease cleavage, and subsequent size-exclusion chromatography (SEC). For protein purification, cells were harvested by centrifugation (4 °C, 2700*g*, 20 min), resuspended in lysis buffer (binding buffer with 0.5 % Triton X-100), and lysed by pulsed ultrasonication (1 s interval, 95 % power, two times 3 min) on ice. The lysate was cleared by centrifugation (40 min, 38,720*g*, 4 °C) and filtration (0.45 μm). For purification, the filtrate was loaded on a His-trap affinity column (5 ml, GE-Healthcare) pre-equilibrated with binding buffer (50 mM phosphate buffer, pH 7.4, 500 mM NaCl, 20 mM imidazole) on an ÄKTA or Bio-Rad system. The loaded column was washed with binding buffer and the protein was eluted by gradient elution with elution buffer (binding buffer with 500 mM imidazole). The fractions were concentrated by centrifugation (4500*g*, 4 °C) using a centrifugal filter unit (Amicon Ultra-15, Merck Millipore Ltd Tullagreen, Carrigtwohill Co Cork Ireland, 10 kDa cut-off). For cleavage, SUMO-protease was added at a molar ratio of 1:100 and incubated overnight at 4 °C. SEC was done using a HiLoad 26/600 Superdex 75 pg, GE Healthcare column equilibrated with 50 mM phosphate buffer pH 7.4. The collected fractions were pooled and concentrated to 1 to 2 mM and stored at −80 °C. Quality and monodispersity of the samples was verified by HPLC SEC (Superdex 75 26/200 GL (GE Healthcare)) coupled to multiangle light scattering and performed on a LC20 prominence HPLC system equipped with a refractive index detector RIF-10A, a photodiode array detector SPD-M20A (Shimadzu), and a multiangle light scattering Heleos Dawn8+ with QELY detector (Wyatt Technology). The column was equilibrated with running buffer (PBS with 200 mM NaCl (pH 7.4)). Experiments were performed at a flow rate of 0.75 ml min^−1^ and 25 °C and resulting data were analyzed using the ASTRA 6 software (Wyatt Technology). All samples were filtered through an Ultrafree-MC filter with a pore size of 0.1 μm (Merck Millipore) and 80 μg of protein was loaded per run.

### SPR spectroscopy

SPR experiments were performed with the BiacoreT200 instrument (GE Healthcare). Human native MPO was biotinylated using the EZ-Link Sulfo-NHS-LC-Biotinylation kit (Thermo Fischer Scientific) and was immobilized on a CAP sensor chip (Cytiva) using the Biotin CAPture kit according to the manufacturer’s protocol (Cytiva). Biotinylated MPO in 50 mM phosphate buffer pH 7.4 was immobilized at a concentration of 2.5 ng/μl and a flow rate of 30 μl/min to a density of 500 resonance unit (RU) on flow cell 2. Flow cell 1 served as a reference surface. Single cycle kinetic experiments were performed using increasing concentrations of SPIN variants (between 2 and 64 nM) in running buffer (PBS, 0.05% Tween, 0.1% bovine serum albumin). Association times were 600 s, dissociation times were 600 s, and the flow rate was set to 30 μl/min. To determine the equilibrium dissociation constant *K*_*D*_, equilibrium response units the data were either fitted to a 1:1 binding model (SPIN-*truncated*) or a two-state transition model (SPIN-*aureus*), which assumes a rebinding event. The R_max_ values were set to constant.

### Differential scanning calorimetry

Differential scanning calorimetry (DSC) experiments were performed with a MicroCal PEAQ-DSC Automated (Malvern Panalytical Ltd) equipped with an autosampler for 96-well plates and controlled by the MicroCal PEAQ-DSC software (Malvern Panalytical Ltd) (cell volume: 130 μl). Samples were measured over a temperature range of 20 to 90 °C with a heating scan rate of 90 °C h^−1^, cooled to 20 °C, and rescanned with the same settings. Two micromolar MPO alone or with 8 μM SPIN-*aureus* or SPIN-*truncated* were preincubated in buffer for 1 h at room temperature (RT). Buffer used were phosphate citrate buffer pH 5 or phosphate buffer pH 7.4, the rescan was used for baseline correction. Data analysis was performed with the MicroCal PEAQ-DSC software using a non–two-state equilibrium unfolding model.

### ITC

ITC experiments were performed with a Microcal PEAQ-ITC Automated (Malvern Panalytical Ltd) equipped with an autosampler for 96-well plates and controlled by the MicroCal PEAQ-ITC software. All samples were diluted in 50 mM phosphate buffer pH 7.4 or pH 5, 10 μM MPO was in the cell, and 150 μM SPIN variants were used as titrant with 19 injections of 3 μl. Data analysis was performed using the Microcal PEAQ-ITC analysis software.

### Steady state and pre–steady state kinetics

For all steady and transient state experiments with the MPO–SPIN complex, the samples were preincubated with equimolar or 2-fold excess of SPIN-*aureus* for at least 1 h at RT. The apparent steady-state kinetic parameters of MPO with and without inhibition by SPIN-*aureus* with H_2_O_2_ and bromide or chloride were determined by monitoring the decrease in absorbance of MCD at 290 nm (E_290 nm_ = 19.9 mM^−1^ cm^−1^) upon reaction of MCD with NaBr using a stirred cuvette and a scanning photometer (Cary 60 spectrophotometer, Agilent Technology). A reaction consisted of 1 ml of 50 mM phosphate–citrate buffer, pH 5.0, and 100 μM MCD, H_2_O_2_ concentrations were varied between 50 and 200 μM, bromide concentrations between 1 and 25 mM, and chloride between 50 and 200 mM. The reaction was initiated with 100 nM MPO or MPO–SPIN-*aureus* complex; all reactions were carried out in triplicates at 25 °C. The initial rate of reaction (*v*_o_) was obtained from the slope between 20 and 40 s; the kinetic parameters were calculated according to the follows:

$$\frac{v_{o}}{E_{0}}=\frac{k_{cat}\left[\text{AB}\right]}{K_{A}\left[B\right]+K_{B}\left[A\right]+\left[\text{AB}\right]}$$

which describes a ping pong bi-bi steady-state kinetic mechanism with *E*_*0*_ representing the enzyme concentration, *K*_A_ and *K*_B_ are the Michaelis constants for bromide (A) and H_2_O_2_ (B), and *k*_cat_ is the turnover number at saturating concentration. Time dependency of the inhibition of MPO was determined by measuring the MCD activity after defined incubation at RT (time points 5 min to 2 h) or by addition of SPIN-*aureus* to MPO after initiation of the reaction (time points 10 s to 1 min) and determining the change in MCD oxidation in 3 s increments at specific time points.

All transient state experiments were conducted with a stopped-flow apparatus (SX-18MV or pi-star equipped with diode array detector or a monochromator) from Applied Photophysics. All measurements were performed at 25 °C. For single wavelength measurements, a minimum of three repeats were performed for each ligand or substrate concentration. Typically, 2 μM MPO or MPO–SPIN-*aureus* complex (5 mM phosphate buffer, pH 7.0) was mixed with at least a 5-fold excess of ligand or substrate in 100 mM buffer (phosphate buffer pH 7 or 7.4 or phosphate-citrate buffer pH 5). The reaction was monitored at distinct wavelength and pH (cyanide: 455 nm, pH 7, nitrite: 455 nm, pH 5, H_2_O_2_: 430 nm, pH 7).

### Crystallization and structure refinement

Native human MPO (10 mg ml^−1^) was incubated for 1 h or overnight with stoichiometric concentrations of SPIN-*aureus* or SPIN-*truncated* in 50 mM phosphate buffer pH 7. Crystallization experiments were performed using SWISSCI MRC 3-well crystallization plates (Molecular Dimensions) adopting the vapor diffusion method. Crystallization drops were set using a Mosquito LCP (TTP Labtech, Melbourne Science Park). The MPO–SPIN-*aureus* complex crystalized in 8% (*w/V)* PEG 20000, 0.1 M BICINE pH 9, 0.5% (*V/V)* Dioxane. The SPIN-*truncated* complex crystallized in 8.5% (*w/V)* PEG 20000, 0.1 M BICINE pH 9, 2.0% (*V/V)* Dioxane. The reservoir was filled with 40 μl crystallization solution. Single drops were set up with a ratio of 100:150, 150:150, and 200:150 protein(nl):crystallization(nl). Crystallization plates were sealed and stored at 22 °C. For cryo-protection, the crystallization conditions were supplemented with 25 % (*V/V)* MNP. All crystals were harvested using cryo-loops and flash-vitrified in liquid nitrogen. Datasets were collected at beamline ID23-2 (48) and at ERSF (European Radiation Synchrotron Facility, Grenoble, France). Data for MPO–SPIN-*aureus* and MPO-SPIN-*truncated* cocrystals (PDB ID: 7QZR and 7Z53) were indexed and integrated with *XDS* (49), the space group was determined with *POINTLESS* (50) and scaled with *AIMLESS* (51), all within the *autoPROC* data processing pipeline (52). Rfree flags for all datasets were created at this stage corresponding to 5% of the measured reflections for each dataset. STARANISO (53) was used for anisotropic cut-off of the merged intensity data. The phase problem for the MPO–SPIN-*aureus* structure was solved by molecular replacement using Phaser-MR (54) taking PDB structure 5UZU of recombinant MPO in complex with SPIN-*aureus*. The phase problem for the MPO-SPIN-*truncated* cocrystals was solved by molecular replacement using 7QZR as a search model and MORDA (55), followed by automated model building with ARP/wARP (56, 57). Data collection and processing statistics are listed in Table 6. Matthews coefficient calculations (58) indicated the presence of one tetramer per asymmetric unit in the MPO–SPIN-*aureus* cocrystals and four tetramers in the MPO-SPIN-*truncated* cocrystal. The models were further improved by iterative model building using maximum likelihood refinement with phenix.refine (59) (with flags set for individual *b*-factor refinement, TLS, and occupancy refinement), manual model building by SSM superpositioning (60) and COOT (61). Models were further optimized by automated model building using PBD-redo (62). Final refinement rounds were performed with *BUSTER* (63, 64) (correcting for wavelength and form factor, with flags set TLS refinement and occupancy refinement). *Grade* (65) was used to generate optimized restraints for the heme b cofactor and refinement was carried out with hydrogen atoms (at zero occupancy) added to the model using aB_hydrogenate within the BUSTER-TNT package. The final models were validated with Molprobity (66). Figures were prepared with PYMOL (http://www.pymol.org/).

**Table 6:** Data collection and refinement statistics for SPIN structures

PDB entry	SPIN-*aureus*		SPIN-truncated	
	7QZR	−	7Z53	−
	*autoPROC*/*STARANISO*	*autoPROC*/*AIMLESS*	*autoPROC*/*STARANISO*	*autoPROC*/*AIMLESS*
Data collection				
Synchrotron	ESRF (Grenoble – France)		ESRF (Grenoble – France)	
Beamline	ID23–2		ID23–2	
Wavelength (Å)	0.873		0.873	
Resolution range (Å)^a^	19.81–2.18 (2.33–2.18)	19.81–2.28 (2.32–2.28)	166.56–2.28 (2.44–2.28)	166.56–2.41 (2.46–2.41)
Space group	*P* 4_3_ 2_1_ 2		*P* 2_1_ 2_1_ 2	
Unit cell				
*a*, *b*, *c* (Å)	112.09, 112.09, 249.95		257.02, 157.20, 166.56	
*α*, *β*, *γ* (^o^)	90, 90, 90		90, 90, 90	
Total no. reflections	5,25,797 (18,209)	5,51,475 (19,365)	1 4,97,194 (63,791)	15,93,333 (81,424)
No. unique reflections	68,945 (3448)	72,785 (3551)	2,42,698 (9894)	2,57,787 (12,769)
Multiplicity	7.6 (5.3)	7.6 (5.5)	6.2 (6.4)	6.2 (6.4)
Completeness spherical (%)	82.4 (23.5)	99.9 (99.9)	79.3 (17.9)	99.9 (100.0)
Completeness ellipsoidal (%)	92.9 (48.4)	−	93.1 (58.8)	−
*<I*/σ(*I*)>	6.2 (1.6)	5.9 (1.1)	4.1 (1.5)	4.0 (1.2)
*R*_merge_ (%)^b^	28.0 (122.0)	29.2 (187.0)	36.2 (169.7)	38.2 (211.7)
*CC*_1/2_ (%)^c^	98.8 (51.2)	98.8 (38.9)	96.4 (49.4)	96.4 (38.3)

Refinement				
*R*_cryst_ (%)^d^	20.38 (27.12)	−	20.28 (27.71)	−
*R*_free_ (%)^e^	24.38 (27.82)	−	23.74 (31.63)	−
Number of non-H atoms				
Protein	10,267	−	40,134	−
Ligands	706	−	2916	−
Waters	526	−	1064	−
RMSD bonds (Å)^f^	0.009	−	0.008	−
RMSD angles (^o^)	0.98	−	0.99	−
Ramachandran plot		−		−
Most favored (%)	97.22	−	97.40	−
Outliers (%)	0.24	−	0.12	−
Rotamer outliers (%)	0.89	−	1.00	−
Clash score^g^	1.23	−	1.50	−
MolProbity score^h^	0.99	−	1.01	−
*B*-factors (Å^2^)				
Protein	32.98	−	42.18	−
Ligands/ions	39.03	−	50.81	−
Waters	29.80	−	32.33	−

a Information in parenthesis refers to the last resolution shell.

b *R*_merge_ = Σ_h_Σ_l_ |*I*_hl_ − <*I*_h_>|/Σ_h_Σ_l_ <*I*_h_>, where *I*_hl_ is the *I*th observation of reflection h and <*I*_h_>.

c *CC*_1/2_ as described in Karplus & Diederichs (2012). Science, 336(6084): 1030–1033.

d *R*_cryst_ = Σ_h_‖*F*_obs(h)_| − |*F*_cal(h)_‖/Σ_h_|*F*_obs(h)_, where *F*_obs(H)_ − *F*_cal(h)_ are the observed and calculated structure factors for reflection *h*, respectively.

e *R*_free_ was calculated the same way as R_factor_ but using only 5% of the reflections which were selected randomly and omitted from refinement.

f RMSD, root mean square deviation.

Chemicalize was used for prediction of Van der Waals volume (November 2021) shown in Figure 5, https://chemicalize.com developed by ChemAxon (http://www.chemaxon.com). UCSF Chimera (67) and PISA (68) were used for prediction of hydrogen bonds, salt bridges, and analysis of the interfaces. In UCSF Chimera hydrogens bonds parameters were set to 0.6 Å distance and 45° angle deviation.

## Data availability

The data for the described crystal structures is available under the PDB accession codes 7QZR and 7Z53, all other data is contained within the article.

## Supporting information

This article contains supporting information.

## Conflict of interest

The authors declare that they have no conflicts of interest with the contents of this article.
