# Supplementary material for: The staphylococcal inhibitory protein SPIN binds to human myeloperoxidase with picomolar affinity but only dampens halide oxidation
Source: J Biol Chem. 2022 Sep 21;298(11):102514. doi: 10.1016/j.jbc.2022.102514 (PMC9615034; doi:10.1016/j.jbc.2022.102514)
Supplement: Supplemental data [file mmc1.docx]

**SUPPORTING INFORMATION**

**The staphylococcal inhibitory protein SPIN binds to human myeloperoxidase with picomolar affinity but only dampens halide oxidation**

Urban Leitgeb1, Paul G. Furtmüller1, Stefan Hofbauer1, Jose A. Brito2, Christian Obinger1, Vera Pfanzagl1*

*to whom correspondence should be addressed

1University of Natural Resources and Life Sciences, Vienna, Department of Chemistry, Institute of Biochemistry, Muthgasse 18, 1190 Vienna, Austria

2 Universidade Nova de Lisboa, Instituto de Tecnologia Química e Biológica António Xavier, 2780-157 Oeiras, Portugal.

**
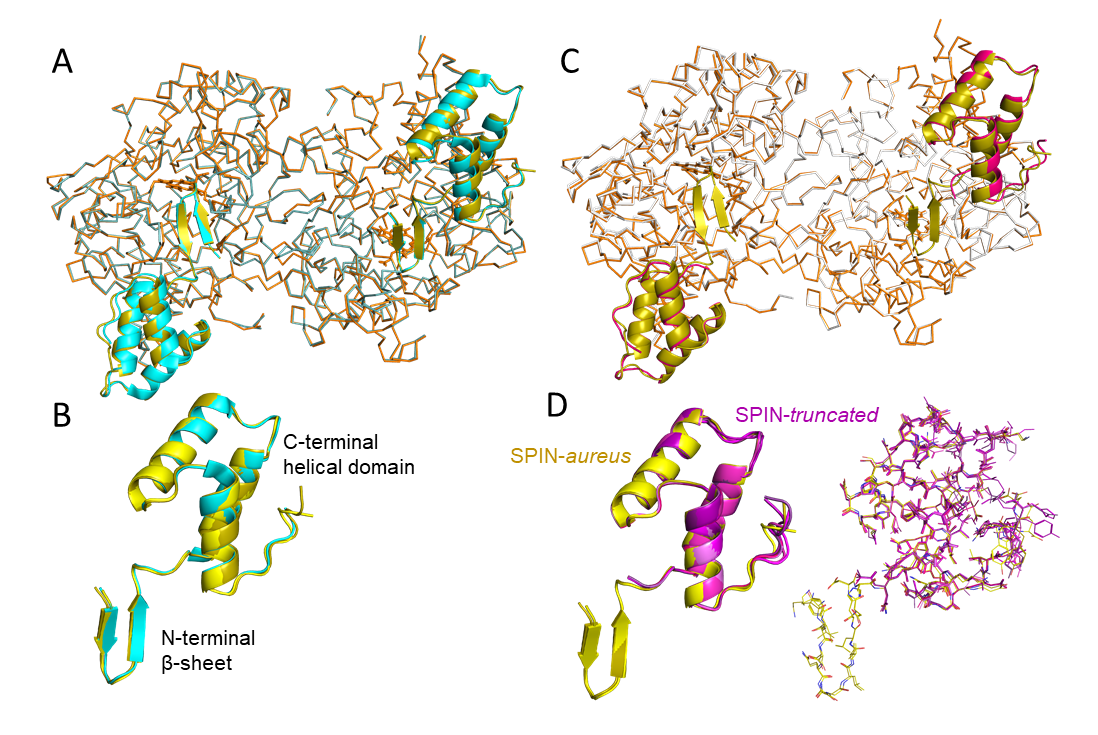
**

Figure S1

Comparison of the co-crystal structures of rMPO-SPIN-*aureus* (PDB: 5UZU), MPO-SPIN-*aureus* (PDB: 7QZR) and MPO-SPIN-*truncated* (PDB:7Z53). Overlay of the full complex of MPO-SPIN-*aureus* and SPIN-*aureus* alone with rMPO-SPIN-*aureus* (A & B) and MPO-SPIN-*truncated* (C & D) are a representative heterotetramer of MPO-SPIN-*truncated* shown in C and the monomeric rMPO-SPIN-*aureus* complex shown twice in A as overlay with the dimeric native MPO-SPIN-*aureus* complex. MPO is depicted as ribbons (5UZU in cyan, MPO-SPIN-*aureus* in orange, MPO-SPIN-*truncated* in white). SPIN variants are shown in cartoon or line representation (SPIN-*aureus* in 5UZU in cyan, SPIN-*aureus* in 7QZR in gold shades and SPIN-*truncated* in shades of magenta.

**
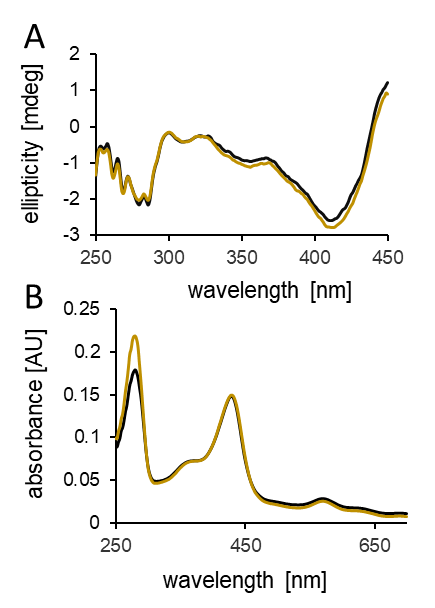
**

Figure S2

Comparison of ECD and UV-vis spectra of MPO alone (black) and in complex with SPIN-*aureus* (gold) in 50 mM Phosphate buffer, pH 7.4.

**
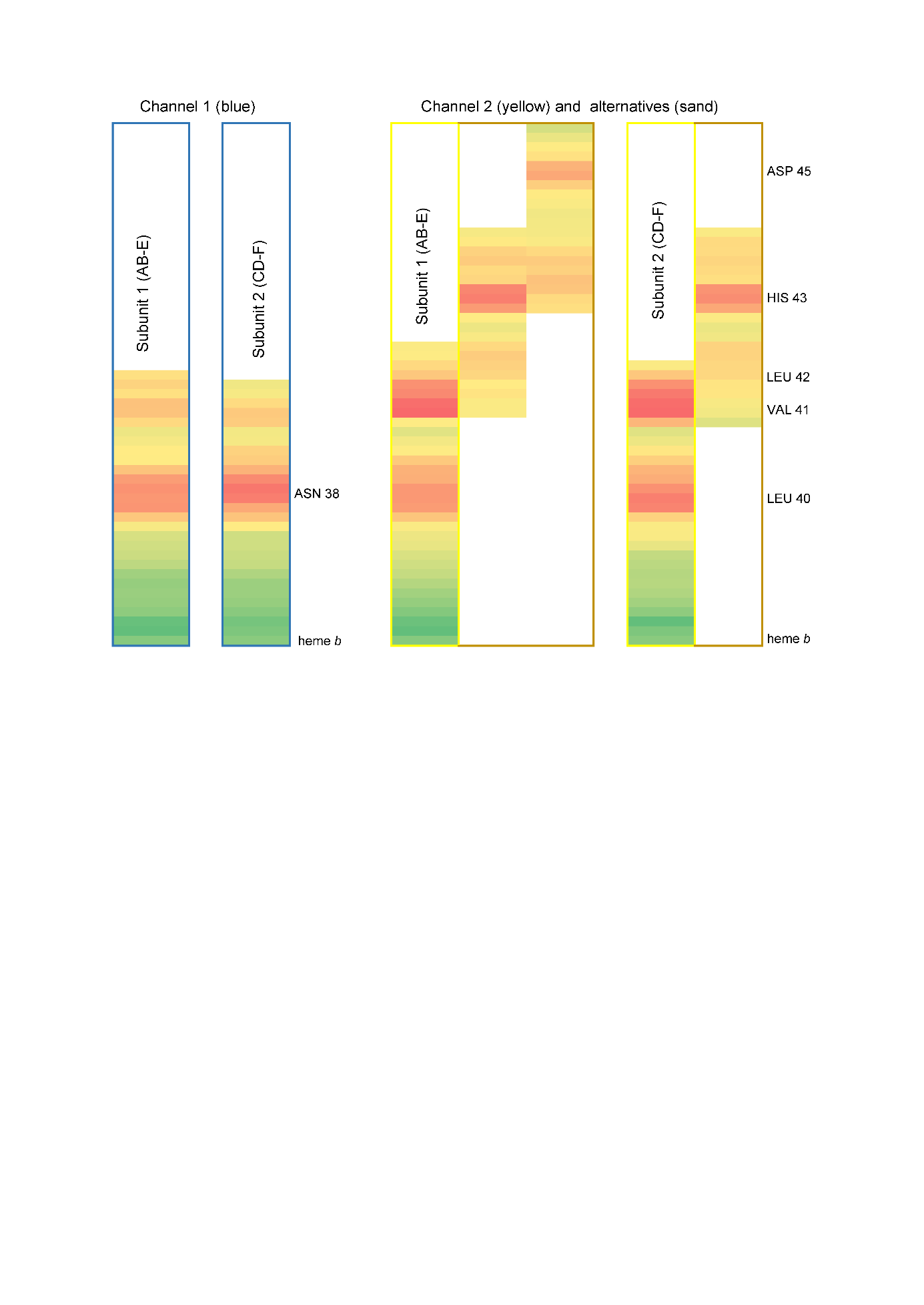
**

Figure S3

Heat map of the channels shown in Figure 2 and calculated by CAVER3.0 for the MPO-SPIN-*aureus* co-crystal structure. Red indicates a small channel radius, i.e bottleneck positions. Relevant residues of SPIN-*aureus* are indicated on the side, the boxes are coloured to match the channel colours in Figure 1.

**
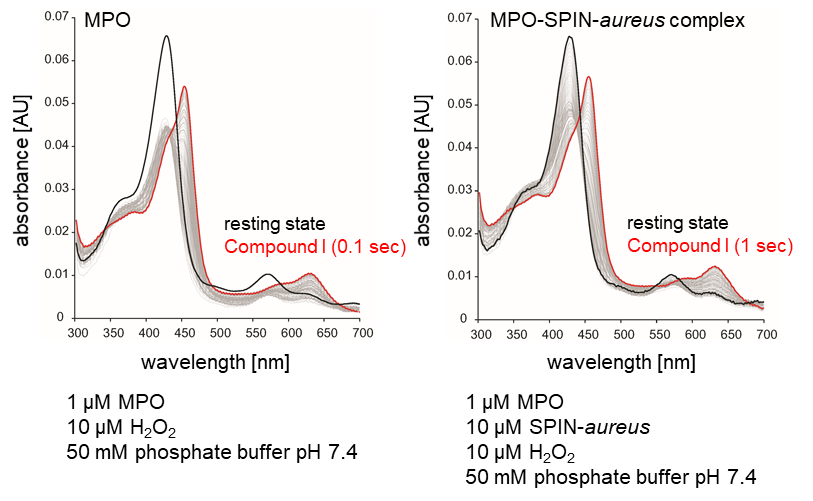
**

Figure S4

Comparison of the UV-vis spectral changes of MPO alone (left) and in complex with SPIN-*aureus* (right) during the reaction with 10 µM H_2_O_2_ in 50 mM phosphate buffer, pH 7.4. The initial spectrum of the resting state is shown in black, intermediate spectra are shown in grey and the final spectrum obtained after 0.1 and 1 sec respectively is shown in red

**Table S1**: detailed PISA analysis of the interface of SPIN-*aureus* with native MPO (PDB: 7QZR) or recombinant MPO (PDB: 5UZU) and SPIN-*truncated* with native MPO (PDB:7Z53)

|  | SPIN-*aureus* | | MPO | | SPIN-*truncated* | | MPO | |
| --- | --- | --- | --- | --- | --- | --- | --- | --- |
| # Residues -all | 30 | 42.9% | 51 / 50 | 8.8 - 8.9% | 17 | 30.85 ± 2.12 % | 21 | 4.45 ± 0.09 |
| - C-terminal D | 17 |  | 18 | 3.7% |  |  |  |  |
| - N-terminal D | 13 |  | 36 | 6.3% |  |  |  |  |
| rMPO-SPIN-*aureus* (5UZU) | 31 | 45.6% | 55 | 9.5% |  |  |  |  |
| Interface Area [A] -all | 1465 | 29.5% | 1404 | 6.1% | 669 ± 24 | 16.75 ± 0.66% | 645 ± 21 | 2.80 ± 0.09% |
| - C-terminal D | 675 |  | 655 | 2.8.7% |  |  |  |  |
| - N-terminal D | 850 |  | 798 | 6% |  |  |  |  |
| rMPO-SPIN aureus (5UZU) | 1589 | 31.7% | 1495 | 6.6% |  |  |  |  |
| ΔiG* gain [kcal/mol] -all | -4.5 | 8.8% | -7.7 | 1.5% | -3.06 ± 0.32 | 7.71 ± 0.67% | 0.89 ± 0.39 | 2.80 ± 0.10% |
| - C-terminal D | -2.4 | 5.3% | -1.8 | 0.4% |  |  |  |  |
| - N-terminal D | -2.2 | 25.4% | -6.3 | 1.2% |  |  |  |  |
| rMPO-SPIN-*aureus* (5UZU) | -4.8 | 9.7% | -5.9 | 1.1% |  |  |  |  |
| average gain | --2.2 | 4.3% | -1-2 | 0.2% | -0.99 ± 0.24 | 2.44 ± 0.63% | -1.54 ± 0.09 | 0.39 ± 0.03% |
| - C-terminal D | -0.8 | 1.8% | -0.9 | 0.2% |  |  |  |  |
| - N-terminal D | -2.0 | 23.0% | -1.5 | 0.2% |  |  |  |  |
| rMPO-SPIN-*aureus* (5UZU) | -2.9 | 5.90% | -3.2 | 0.6% |  |  |  |  |
| P-value for ΔiG** | 0.272 |  | 0.070 |  | 0.21 ± 0.04 |  | 0.60 ± 0.05 |  |
| - C-terminal D | 0.276 |  | 0.360 |  |  |  |  |  |
| - N-terminal D | 0.616 |  | 0.0475 |  |  |  |  |  |
| rMPO-SPIN-*aureus* (5UZU) | 0.32 |  | 0.245 |  |  |  |  |  |

*ΔiG indicates the solvation free energy gain due to formation of the interface, in kcal/mol, calculated as the difference in total solvation energies of isolated and interfacing structures. Negative ΔiG corresponds to hydrophobic interfaces, or positive protein affinity without considering hydrogen bonds and salt bridges.

**ΔiG P-value indicates the P-value of the observed solvation free energy gain and is a measure for the probability of getting a lower than observed ΔiG, when the interface atoms are picked randomly from the protein surface. The P-value is a measure of interface specificity and P<0.5 indicates a higher than average hydrophobicity.

**Table S2**: CAVER analysis of access channels in the co-crystal structure of MPO-SPIN-*aureus* shown in Figure 2.

|  | channel | cost | bottleneck radius [Å] | length [Å] | bottleneck residues |
| --- | --- | --- | --- | --- | --- |
| subunit 1 (AB-E) | 1 - (blue) | 0.81 | 1.11 | 22.62 | ASN 38 |
|  | 2- (yellow-orange) | 1.22 | 0.92 | 24.96 | LEU 40, VAL 41, LEU 42 |
|  | 3 -(yellow) | 1.36 | 1.03 | 32.58 | LEU 40, VAL 41, LEU 43, HIS 43 |
|  | 4 - (yellow-sand) | 1.46 | 1.13 | 36.37 | LEU 40, VAL 41, LEU 44, ASP 45 |
| subunit 2 (CD-F) | 1 | 0.82 | 0.99 | 22.15 | ASN 38 |
|  | 2 | 1.13 | 0.94 | 23.62 | LEU 40, VAL 41, LEU 42 |
|  | 3 | 1.41 | 1.03 | 33.44 | LEU 40, VAL 41, LEU 43, HIS 43 |

**Table S3:** Thermostability and enthalpic parameters of MPO alone or in complex with SPIN-*aureus* or SPIN-*truncated* determined by DSC in 50 mM Phosphate buffer, pH 7.4 or Phosphate-Citrate buffer, pH 5

|  | *T*_m_ [°C] | | | ∆*H* [kJ/mol] | | | ∆*H*_VH_ [kJ/mol] | | |
| --- | --- | --- | --- | --- | --- | --- | --- | --- | --- |
| pH | MPO | MPO-SPIN-*aureus* | MPO-SPIN-*truncated* | MPO | MPO-SPIN-*aureus* | MPO-SPIN-*truncated* | MPO | MPO-SPIN-*aureus* | MPO-SPIN-*truncated* |
| 7.4 | 80.3 | 81.9 (+1.6) | 77.3 (-3.0) | 573 | 666 | 451 | 489 | 558 | 420 |
|  | 83.6 | 85.5 (+1.9) | 83.7 (+0.1) | 786 | 804 | 551 | 927 | 1140 | 900 |
|  | 86.8 | 88.2 (+1.5) | 87.6 (+0.9) | 1660 | 2250 | 1450 | 968 | 1170 | 870 |
| 5 | 78.8 | 86.4 (+7.7) | 78.9 (+0.2) | 260 | 718 | 381 | 480 | 572 | 457 |
|  | 83.7 | 89.9 (+6.2) | 84.2 (+0.5) | 460 | 891 | 610 | 1030 | 1130 | 933 |
|  | 87.4 | 92.4 (+5.0) | 87.7 (+0.3) | 2450 | 2320 | 2280 | 890 | 1100 | 915 |
